# TUBA1C orchestrates the immunosuppressive tumor microenvironment and resistance to immune checkpoint blockade in clear cell renal cell carcinoma

**DOI:** 10.3389/fimmu.2024.1457691

**Published:** 2024-09-05

**Authors:** Junyi Li, Meixue Chen, Ming Tong, Qingfei Cao

**Affiliations:** ^1^ Department of Urology, The First Affiliated Hospital of Jinzhou Medical University, Jinzhou, Liaoning, China; ^2^ The First Clinical Medical College, Jinzhou Medical University, Jinzhou, Liaoning, China; ^3^ Department of Pediatric, The First Affiliated Hospital of Jinzhou Medical University, Jinzhou, Liaoning, China

**Keywords:** clear cell renal cell carcinoma, single cell RNA sequencing, tubulin alpha 1c, immune checkpoint blockade, immunotherapy resistance, immunosuppressive tumor microenvironment, prognostic biomarker

## Abstract

**Background:**

Clear cell renal cell carcinoma (ccRCC) poses substantial treatment challenges, especially in advanced stages where the efficacy of immune checkpoint blockade (ICB) therapy varies significantly. Elevated expression of the oncogene *TUBA1C* has been correlated with poor prognosis in various cancers, however, its role in ccRCC is unclear, especially concerning ICB resistance.

**Methods:**

Single-cell analysis was used to examine gene expression variations in malignant cells post-ICB therapy. This included investigating TUBA1C expression across different ICB response groups and its relationship with CD274. A general module of action was identified through pan-cancer and pan-tissue analysis. TUBA1C expression and its association with clinical characteristics and prognosis was further validated. Multiple algorithms were employed to explore immune cell infiltration levels, and the DepMap database was utilized to assess gene dependency and mutation status in kidney cancer cell lines. The *in silico* knockout of TUBA1C was performed using deep learning model, complemented by immunohistochemical assays, clinical cohort and functional assays validations.

**Results:**

TUBA1C expression is elevated in malignant cells following ICB therapy and is correlated with ICB resistance in ccRCC. High TUBA1C expression activates PI3K/AKT pathway and is associated with increased infiltration of regulatory T cells and myeloid-derived suppressor cells, which contributes to an immunosuppressive microenvironment in ccRCC. Patients with high TUBA1C expression exhibit a greater tumor mutation burden and increased genetic variation, which causes a worse prognosis. Additionally, TUBA1C dependency and its effects were evident in kidney cancer cell lines, where mutations conferred resistance to anti-PD-L1 therapy. *In silico* knockout analyses indicated that treatment targeting TUBA1C shifted malignant cells to a state responsive to ICB therapy. Immunohistochemistry, RT-qPCR and clinical cohort validation further confirmed that TUBA1C expression was upregulated and contributed to poorer outcome in ccRCC. Finaly, wound healing and CCK-8 assays demonstrated the potent oncogenic function of TUBA1C.

**Conclusions:**

TUBA1C is a pivotal regulator in ccRCC, affecting both disease progression and the effectiveness of ICB therapy by fostering an immunosuppressive microenvironment mediated by the PI3K/AKT pathway. Additionally, TUBA1C holds promise, both as a prognostic biomarker and a therapeutic target, for enhancing responsiveness to ICB.

## 1 Introduction

Renal cell carcinoma (RCC) is a significant cause of mortality among kidney diseases, with clear cell RCC (ccRCC) being the most prevalent pathological subtype (1). Although surgical resection is the primary mode of treatment in the early stages of the disease, many patients with ccRCC present with do not display clinically significant symptoms early on, leading to a delay in treatment (2). Accordingly, approximately 30% of ccRCC patients are diagnosed at an advanced stage without the option for surgery (2, 3). Furthermore, ccRCC patient populations have become increasingly younger in recent years, characterized by high rates of metastasis and mortality (4). Collectively, these factors pose challenges to treatment approaches.

A wide array of treatments are currently available for enhancing the prognosis of ccRCC patients, including targeted therapy, immune checkpoint blockade (ICB), and anti-angiogenic therapy, among others. The integration of ICB with other therapeutic strategies has significantly extended patient survival. However, the five-year survival rate for patients with advanced-stage disease remains unsatisfactory (3, 5). Under physiological conditions, immune checkpoint pathways play a crucial role in modulating the processes of infection and tumorigenesis and are intricately involved in tumor immune evasion mechanisms (6). Additionally, the efficacy of response to treatments targeting these pathways is across a range of solid tumors generally limited (7). Despite widespread resistance to ICB therapies, the genetic and molecular basis of such resistance remains largely unexplored (8, 9). Resistance to ICB and tumor metastasis involves complex intercellular interactions and evolutionary processes. There is significant heterogeneity in gene expression among cells within tumors, which greatly affects the makeup of the tumor microenvironment (TME). Additionally, differences in cell infiltration levels in the TME play a critical role in tumor progression, affecting immune adaptation and evasion (10, 11). Studies have confirmed that ccRCC is among the cancer types most infiltrated by immune and vascular elements (12, 13). Moreover, substantial evidence supports that the TME is among the most critical factors impacting the response and resistance to ICB. The immune cells, stromal cells, metabolic status, and microbial components in the TME mutually influence each other (6, 14). Therefore, understanding the genetic and molecular features of the TME is essential for assessing patient prognosis and developing effective targeted treatments in ccRCC.

Multiple studies have revealed the oncogenic role of tubulin alpha-1C chain (TUBA1C) in various cancer types. In lung adenocarcinoma, TUBA1C has been identified as a robust prognostic biomarker associated with the abundance of immune cell infiltration (15).Additionally, TUBA1C promotes the progression of bladder cancer via the regulation of the cell cycle (16), and accelerates the progression of gastric cancer by activating the PI3K/AKT and cell cycle-related pathways (17). Overall, TUBA1C exhibits a common function across various cancer types, regulating the PI3K/AKT and cell cycle-related pathways and reshaping the TME, thereby influencing tumor progression and, consequently, leading to poor clinical outcomes.

Integrating high-throughput technologies and computational biology is an effective and innovative approach to explore potential mechanisms and therapeutic targets of ICB resistance, which can accelerate clinical translation and improve patient outcomes (18). In this study, we integrated single-cell RNA sequencing with bulk RNA analysis to explore differences in gene expression at both single-cell and tissue levels post-ICB therapy, as well as variations in cell proportions. We focused primarily on malignant cells in ccRCC to identify potential functionally relevant genes closely associated with the expression of PD-L1, a protein that aid tumor cells evade cytotoxic T cell-mediated destruction (7). We investigated functions of TUBA1C, its impact on tumor progression, and prognostic outcomes, and its association with immune cell infiltration across several cohorts. Additionally, we conducted a bioinformatics analysis using various kidney cancer cell lines to demonstrate the effects of TUBA1C gene function on the cellular phenotypes. Based on the obtained results, we proposed a reasonable hypothesis, namely, that TUBA1C recruits myeloid-derived suppressor cells (MDSCs) and regulatory T cells (Tregs) via the PI3K/AKT pathway, thereby inducing an immunosuppressive phenotype in macrophages and dysfunction in CD8+ T cells. This process reshapes the immunosuppressive tumor microenvironment and mediates ICB resistance in ccRCC. To further confirm the relationship between ICB resistance and TUBA1C expression, mutation data relating to several cancer cell lines were analyzed to explore the sensitivity of the cells to anti-PD-L1 therapy. Additionally, a deep learning model was used to validate the response state of malignant cells at the single-cell level following the *in silico* knockout of TUBA1C. Furthermore, we assessed the protein expression levels of TUBA1C and PD-L1 within ccRCC tissue microarrays (TMAs) to explore their prognostic and clinical significance. Finally, the RNA expression levels and oncogenic functions of TUBA1C were further validated in kidney cancer cell lines. Our aim was to identify a biomarker that can enhance precision-targeted therapy and improve the response to ccRCC immunotherapy.

## 2 Materials and methods

### 2.1 Data collection and filtering

In this study, a single-cell RNA sequencing (scRNA-seq) dataset mined from the Single Cell Portal (https://singlecell.broadinstitute.org/single_cell), encompassing seven ccRCC samples and one renal papillary cell carcinoma (RPCC) sample, was used to explore the cellular landscape under both untreated and ICB-treatment conditions (9). The inclusion criteria were employed to ensure the integrity and applicability of the dataset for downstream analysis included: (1) scRNA-seq data derived from patients diagnosed with ccRCC; (2) samples were biopsy specimens obtained directly from renal tumors; (3) samples from patients who had been treated with tyrosine kinase inhibitors (TKI) before sample collection were excluded. For the analysis of ICB treatment effects on ccRCC, RNA-seq datasets from patients enrolled in the CheckMate 009, 010, and 025 trials were included, as detailed in the study by Braun et al. (19). The RNA-seq data were subjected to strict selection criteria, namely, patients who had received mTOR inhibitor therapy, those who received combination treatment with TKI and ICB, those who had not received ICB treatment, or those whose data on overall response rates were incomplete were excluded. These criteria were applied to avoid the impact of confounding factors and to precisely assess gene function in treatment responses. Additionally, gene expression profiles and associated clinical information from both TCGA Pan-Cancer Atlas and the TCGA-KIRC dataset, supplemented by the GTEx pan-tissue dataset, were also included, all of which were accessed through the UCSC Xena platform (https://xena.ucsc.edu/). The E-MTAB-1980 cohort, serving as an additional resource, was obtained from the ArrayExpress database (https://www.ebi.ac.uk/arrayexpress).To elucidate candidate gene dependencies and effects within the studied populations, data was obtained from The Cancer Dependency Map Project (DepMap, https://depmap.org/portal/), an initiative by the Broad Institute aimed at systematically identifying functionally relevant genes across a broad spectrum of cancer cell lines.

### 2.2 Comprehensive scRNA-seq data processing

For the processing of scRNA-seq data, the ‘Seurat’ R package was employed. Initially, cells were subjected to a stringent filtering process, requiring gene expression levels to exceed 400 but not surpass 6,000, raw read counts to exceed 1000, and mitochondrial gene expression to remain below 20%. Following this, the raw read counts were normalized to balance variations in sequencing depth, and the top 2,000 genes exhibiting the highest variability were identified for subsequent analysis. Following scaling, principal component analysis (PCA) was employed to identify genes with the most significant contribution to data variability, these genes were specifically selected due to their significant contribution to data variability. The ‘Harmony’ integration algorithm was applied to address batch effects across samples and thus ensure that subsequent analyses were not confounded by non-biological sources of variation. Cell Clustering was achieved using the *k*-shared nearest neighbors (SNN) algorithm, to identify discrete cell populations within the complex ccRCC microenvironment. Visualization of the distinct cell clusters was accomplished through Uniform Manifold Approximation and Projection (UMAP) plots, providing a comprehensive overview of cellular heterogeneity. The final step involved the annotation of each cell cluster, which was achieved by leveraging gene markers identified in previous studies (9, 20), and allowed for the precise characterization of cell types within the ccRCC microenvironment.

### 2.3 Single-cell copy number variation analysis

The ‘infercnv’ R package ( https://github.com/broadinstitute/infercnv ) was utilized to conduct a single-cell copy number variation (CNV) analysis on epithelial cells derived from ccRCC samples. This analysis aimed to identify malignant cells within the ccRCC microenvironment by comparing their CNV profiles to those of macrophages and endothelial cells, which served as references (21). Specifically, chromosomal CNVs, including amplifications and deletions, were analyzed within each epithelial subcluster. The ‘infercnv::run’ function was configured with a cutoff value of 0.1 and ‘denoise=TRUE’ to enhance the accuracy of CNV detection. The outcome of this analysis was visualized through a heatmap, depicting the CNV levels across 23 chromosomes in epithelial subclusters relative to the reference cell populations. From this CNV landscape, the malignancy of tumor cells was inferred, providing insights into the genomic instability characteristics of ccRCC.

### 2.4 Analysis of cell differentiation trajectories

To elucidate the cell differentiation pathways within the single-cell dataset, pseudotime analysis was employed using the ‘monocle’ R package (22). This algorithm is designed to estimate size factors and dispersions for the input data, enabling the selective filtration of genes with low expression levels. Subsequent dimensionality reduction is achieved through the ‘DDRTree’ algorithm, facilitating a more refined analysis. Crucially, ‘monocle’ leverages these processed data to construct a comprehensive model of cell differentiation trajectories, mapping distinct cellular states along the pseudotime dimension based on their gene expression profiles. This approach not only delineates the progression of cell states but also provides invaluable insights into the dynamic regulatory mechanisms underpinning cell differentiation.

### 2.5 Intercellular interactions analysis

To investigate the nuances of intercellular communication across various treatment groups as well as pinpoint the pivotal cell clusters responsive to ICB therapy, ‘CellChat’ R package was employed. Specific intercellular interactions among distinct cell clusters were identified based on information in the ‘CellChatDB.human’ ligand-receptor database (23). This analysis enabled the detection of overexpressed genes and their interactions, further allowing for the elucidation of common pathways previously confirmed in the literature. The differential number and strength of these interactions were visualized through the utilization of circle plots and heatmaps, offering insightful perspectives on dynamic interactions at the cellular level.

### 2.6 Prognostic biomarker identification in ccRCC *via* the ICB therapy cohort

For prognostic biomarker identification, the investigation focused on malignant cells in ccRCC, characterized by significant expression of the PD-L1 on the cell membranes, marking it as a pivotal target for ICB therapy. The ‘FindMarkers’ function was used to identify differentially expressed genes (DEGs) within tumor cells and Tumor-Associated Epithelial Cells (TECs) from scRNA-seq datasets, comparing untreated (NO-ICB) and partial response (ICB-PR) groups. These DEGs were then pooled to capture a comprehensive set of genes showing altered expression in response to ICB therapy. DEGs were characterized based on an average log2 fold change (log2FC) >0.5 and a p-value <0.01. All identified DEGs were subsequently analyzed within the Braun ICB cohort through bulk RNA sequencing. To prioritize our list of genes, both univariate and multivariate cox regression analyses were conducted, aiming to identify prognostic biomarkers within the Braun ICB cohort. All DEGs were identified from malignant ccRCC cells, with PD-L1 serving as a key functional protein mediating tumor progression and immune evasion in these cancer cells. Subsequently, the relationship between prognostic genes and CD274 expression was further explored. Spearman correlation analysis was performed to assess the association between candidate genes and CD274 expression, with a p-value < 0.05 considered statistically significant. Furthermore, the expression levels of correlated genes were examined across different response groups, namely, progressive disease (PD), stable disease (SD), and comprehensive response/partial response (CR/PR).

### 2.7 Comprehensive pan cancer and pan tissue analysis

To investigate the correlation between the expression of TUBA1C and CD274 across various cancers and tissues, a Pearson correlation analysis was performed on the TCGA Pan Cancer cohort (excluding normal and duplicated samples) and the GTEx dataset, which enabled an analysis at both the pan-cancer and pan-tissue levels. These datasets were subsequently integrated to assess the variation in TUBA1C expression between normal and tumor tissues. Additionally, the differences in TUBA1C methylation levels and CNV in different cancers were investigated, and their correlation with TUBA1C expression was examined. Moreover, univariate Cox regression analysis was employed to determine the impact of TUBA1C on clinical outcomes, including overall survival (OS), progression-free interval (PFI), disease-specific survival (DSS), and disease-free interval (DFI). Finally, survival differences were assessed based on median expression levels of TUBA1C within TCGA-KIRC and E-MTAB-1980 cohorts, to validate the prognostic significance of TUBA1C. A detailed analysis of TUBA1C expression levels and their association with clinical features within the two cohorts was conducted to enhance understanding of its prognostic relevance.

### 2.8 Deciphering the role of TUBA1C: KEGG and GO enrichment analysis in TCGA-KIRC cohort

To elucidate the functional implications of TUBA1C, a Spearman correlation analysis was conducted between TUBA1C and all genes within TCGA-KIRC cohort. The resulting correlation coefficients were then ranked and used for Gene Set Enrichment Analysis (GSEA), employing both Kyoto Encyclopedia of Genes and Genomes (KEGG) and Gene Ontology (GO) gene sets sourced from the Molecular Signatures Database (MSigDB, https://www.gsea-msigdb.org/gsea/msigdb/index.jsp). The top 5 enrichment results were subsequently visualized to highlight the most significant functional associations of TUBA1C.

### 2.9 The impact of TUBA1C expression on the tumor mutation burden

Somatic mutation data for TCGA-KIRC cohort were retrieved from TCGA database (https://portal.gdc.cancer.gov/). The ‘maftools’ R package was used for calculating the tumor mutation burden (TMB) and visualizing the relevant data. The investigation focused on assessing the TMB in various TUBA1C expression groups to determine the association between TUBA1C expression levels and the TMB. Finally, each sample was stratified into distinct groups based on the median values of TUBA1C expression and the TMB, aiming to predict the OS of ccRCC patients.

### 2.10 Analyzing hallmark pathway activity in relation to TUBA1C expression variability

To evaluate the influence of TUBA1C expression levels on pathway activities, the gene set activity scores among distinct TUBA1C expression groups, categorized by the median values of TUBA1C expression, were calculated with the ‘GSVA’ R package. This analysis specifically targeted the hallmark pathway gene set. Subsequent analysis of pathway activity variation was conducted using the ‘limma’ R package. Pathways exhibiting an absolute *t*-value >2 and a *p*-value <0.05 were identified as exhibiting significant differences in activity.

### 2.11 Dissecting the association between TUBA1C expression and immune cell infiltration in the TME

The interaction between TUBA1C expression and the infiltration of immune cells in the TME within TCGA-KIRC cohort was investigated with the ‘IOBR’ R package (https://github.com/IOBR/IOBR), a tool for analyzing immune cell infiltration patterns. Furthermore, the ‘ssGSEA’ function was used to assess the infiltration levels of 28 immune cell types across different TUBA1C expression categories based on immune cell marker genes identified in a recent study (24). In addition, various tumor and TME signature scores were calculated, as aggregated by the package. Finally, a correlation analysis was performed to uncover the association between TUBA1C expression and immune cell infiltration, thereby shedding light on how TUBA1C may influence the immune landscape within the TME.

### 2.12 Elucidating the role of TUBA1C in ccRCC immune modulation and drug sensitivity *via* the BEST database

The Biomarker Exploration of Solid Tumors online database was used for visualizing the correlation between TUBA1C expression levels and various immune regulatory modules within distinct ccRCC datasets (25). These modules encompass antigen presentation, immune inhibitors, immune stimulators, chemokines, and receptors. Furthermore, leveraging the BEST database, drug sensitivity was predicted for different TUBA1C expression groups using insights from the Genomics of Drug Sensitivity in Cancer (GDSC) and the Cancer Therapeutics Response Portal (CTRP) databases.

### 2.13 Exploring TUBA1C as a potential therapeutic target in ccRCC

Utilizing the DepMap database, which encompasses over 1,000 cancer cell lines with gene knockouts generated using CRISPR-Cas9 technology, an in-depth investigation of the effects of gene function on cancer cell growth and proliferation was undertaken. This is a comprehensive resource that facilitates the identification of viable therapeutic targets by closely monitoring the behavior of cancer cells post-gene knockout. In the current investigation, the DepMap 23Q2 public dataset was used to scrutinize the effect of TUBA1C gene function on the survival and proliferation of cancer cells, focusing on 26 kidney cancer cell lines. Additionally, somatic mutation data was employed to assess the sensitivity of these cells to anti-PD-L1 therapy in the presence (wild-type category) or absence (mutant category) of TUBA1C mutations. The average dependency of CD274 within the same tissue type was then subjected to further analysis, that is, mutant cell lines in the same tissue category with fewer than two representatives were excluded.

To delve deeper into the influence of TUBA1C on the response to ICB therapy, the “Geneformer” deep learning model was employed for *in silico* treatment analysis (26), simulating gene function and drug resistance. This approach involves the use of the Transformer model as the computational backbone specifically tailored for natural language processing tasks (27). This methodology, which closely followed the protocols outlined in (26), facilitates the *in silico* knockout of genes by parsing and processing gene names, segregating malignant cells into training and testing datasets at an 8:2 ratio according to ICB response. The dataset was processed by tokenizing gene names and ranking the genes by their expression levels in each cell. The cells classified as malignant were then allocated into training and testing sets according to their cellular states. The model was fine-tuned over five epochs using the ICB response as a predictive label, aiming to optimize a balance between predictive accuracy and validation loss. The most effective model was subsequently employed for validation purposes and its efficacy was assessed on the test dataset. Following this, an extensive analysis of the malignant cells was conducted using the fine-tuned model to visualize the cellular embeddings. This process culminated in the selection of average cell embeddings for both the NO-ICB and ICB-PR conditions, serving as the initial and target states for malignant cell analysis. To minimize graphics card memory usage and enhance computational efficiency, 2,000 malignant cells were randomly selected, each of which was subjected to *in silico* knockout targeting 2,048 genes with high expression levels. The genes whose knockout resulted in a shift towards the ICB-PR cell state, based on a false discovery rate (FDR) <0.05, were categorized as potential candidates for enhancing the efficacy of ICB therapy.

### 2.14 Clinical cohort and immunohistochemical staining

The TMA for ccRCC, comprising 150 ccRCC tissue samples and 30 patient-matched adjacent non-tumor tissue samples, was acquired from Shanghai Outdo Biotech (Shanghai, China). The clinical characteristics and survival data for 150 ccRCC patients were used to corroborate the findings of the bioinformatics analyses. Ethical approval for this research was granted by the Institutional Review Board of Shanghai Outdo Biotech (approval number: YB M-05-02). The TMA was heated in an oven at 63°C for 1 h to melt the wax, and then dewaxed through a sequence of washes in an automated stainer—twice in xylene, 15 min each wash, followed by two cycles in 100% alcohol, 7 min each wash, and sequential washes (5 min each) in decreasing alcohol concentrations (90%, 80%, and 70%). Following antigen retrieval in a Target Retrieval Instrument, the slides were cooled to room temperature in distilled water. For primary antibody staining, the slides were first rinsed with phosphate-buffered saline (PBS) and then covered with a solution containing the primary antibody (1:200 dilution). Following three PBS washes, each lasting 1 min, secondary antibody binding and DAB chromogenic staining were performed as per the Autostainer Link 48 manual. Hematoxylin staining was applied for 1 min, followed by a quick dip in 0.25% hydrochloric acid in alcohol (composed of 400 mL of 70% alcohol plus 1 mL of concentrated hydrochloric acid) for 10 s, and a 5-min rinse in tap water. Staining results indicated that the TUBA1C protein was localized to the cytoplasm, while PD-L1 expression was observed on the cell membrane as well as the cytoplasm. The immunohistochemistry results for each sample were independently interpreted by three senior pathologists from the First Affiliated Hospital of Jinzhou Medical University. The most objective interpretation was then selected by a fourth pathologist and the results following further analysis.

### 2.15 Cell culture

The mRNA expression of TUBA1C in normal and tumor cells was investigated using the human ccRCC cell lines 786-O and 769-P and the human renal cell line HK-2. The two ccRCC cell lines were cultured in RPMI-1640 while the HK-2 cells were cultured in DMEM/F-12; both media were supplemented with 10% fetal bovine serum and 1% penicillin–streptomycin. Cultures were maintained at 37°C in a humidified incubator with 5% CO2.

### 2.16 RT-qPCR

RNA was isolated using the RNAeasy Animal RNA Isolation Kit with Spin Column (Beyotime, Shanghai) and reverse-transcribed into cDNA using NovoScript Plus All-in-one 1st Strand cDNA Synthesis SuperMix (gDNA Purge). Subsequently, qPCR was performed with NovoStart SYBR qPCR SuperMix Plus (Novoprotein, Suzhou) on the QuantStudio Three Real-Time PCR System (Thermo Fisher). The sequences of the primers used for TUBA1C amplification are provided in **Supplementary Table 1**.

### 2.17 Plasmid transfection

The TUBA1C-siRNA plasmid was constructed by GeneChem (Shanghai) and subsequently transfected into 786-O and 769-P cells. Plasmid transfection was performed using Lipofectamine 3000, while jetPRIME Versatile DNA/siRNA transfection reagent (Polyplus Transfection, France) was used for siRNA transfection into the respective cancer cell lines. The TUBA1C-siRNA sequences are detailed in **Supplementary Table 1**.

### 2.18 Wound healing assays

Cells were seeded in 6-well plates and, at confluence, a uniform scratch was made across the cell monolayer with a sterile 200-μL pipette tip, and the cells were cultured in serum-free medium. Images of wound closure were captured at 0, 12, and 24 h using an inverted phase-contrast microscope. The wound area was quantified using ImageJ software.

### 2.19 CCK-8 assay

A total of 1,000 cells per well were seeded in a 96-well plate and allowed to completely adhere. The assay was performed using Super-Enhanced Cell Counting Kit-8 (Beyotime, Shanghai) according to the manufacturer’s instructions. Following treatment, 100 μL of fresh medium containing 10 μL of CCK-8 reagent was added to each well, and the cells were further incubated at 37°C for 1.5 h. The optical density at 450 nm (OD450) was measured using a microplate reader.

### 2.20 Statistical analysis

All data processing and statistical analyses were executed using R software (Version 4.3.2). DEGs across different ICB response groups was determined using the Wilcoxon test. We utilized both univariate and multivariate Cox regression analyses to pinpoint prognostic genes, while relationships among various variables were explored through Spearman’s rank and Pearson’s correlation coefficients. Furthermore, we assessed prognostic disparities via Log-rank tests and Kaplan–Meier survival analyses across different TUBA1C expression groups. In this study, a p-value <0.05 was considered statistically significant.

## 3 Results

### 3.1 The landscape of the ccRCC microenvironment following ICB therapy

**Figure 1:**
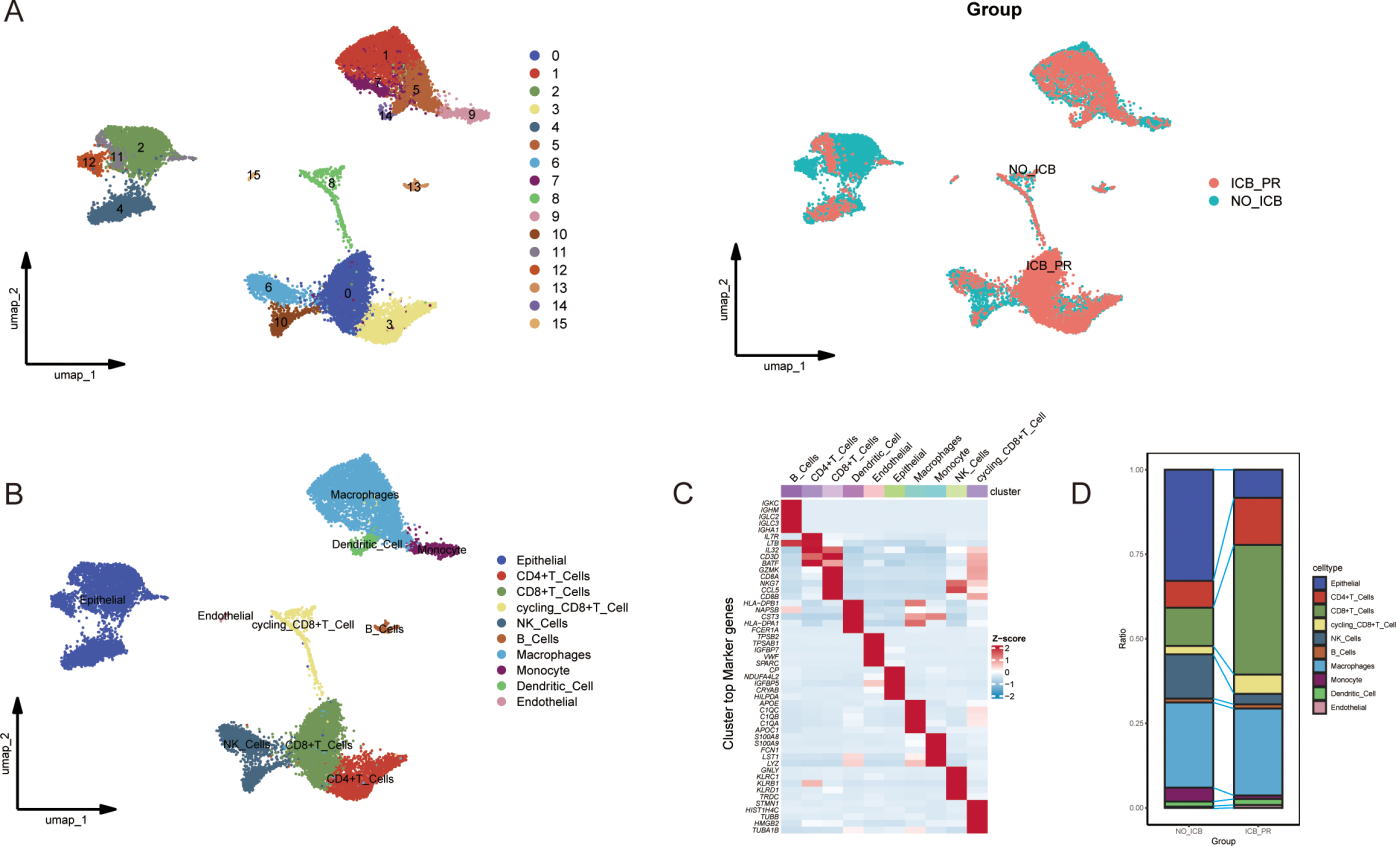
Single-cell atlas of ccRCC with varying responses to ICB. **(A)** UMAP plots illustrating the clustering of cells into 16 distinct groups (left) and differentiated by color to represent the different ICB treatment statuses (right). **(B)** UMAP plots showing the distribution of the 10 major cell types in ccRCC samples from patients with partial response to ICB therapy (ICB-PR) and those not receiving ICB therapy (NO-ICB). **(C)** A heatmap displaying the top five marker genes expressed in each cell cluster. **(D)** A bar plot highlighting significant differences in the prevalence of the 10 major cell types between the ICB-PR and NO-ICB groups in ccRCC. ccRCC, clear cell renal cell carcinoma; UMAP, Uniform Manifold Approximation and Projection; ICB, immune checkpoint blockade; PR, partial response.

To investigate the ccRCC microenvironment at the single-cell level post-ICB therapy, we meticulously filtered the tumor samples for our ICB scRNA-seq analysis. We included two untreated samples and one treated sample showing a partial response, with all biopsy specimens being sourced from primary tumors, while none were from patients who had received TKI therapy, as previously detailed (9). Following the removal of batch effects and rigorous cell quality control, we isolated 18,111 high-quality cells expressing 29,890 genes, which we organized into 16 distinct clusters (**Figure 1A**). These clusters were classified into 10 major cell types characteristic of the ccRCC TME based on the expression of classic cell markers as reported in prior studies (**Figure 1B**). The five most significant marker genes for each cell type are depicted in a heatmap in **Figure 1C**. Further analysis to ascertain the changes in cell proportions post-ICB therapy revealed a significant decrease in the proportions of epithelial cells, natural killer (NK) cells, and monocytes. In contrast, the proportions of CD4+ T cells, CD8+ T cells, and cycling CD8+ T cells were significantly elevated (**Figure 1D**).

### 3.2 The identification of malignant cells

**Figure 2:**
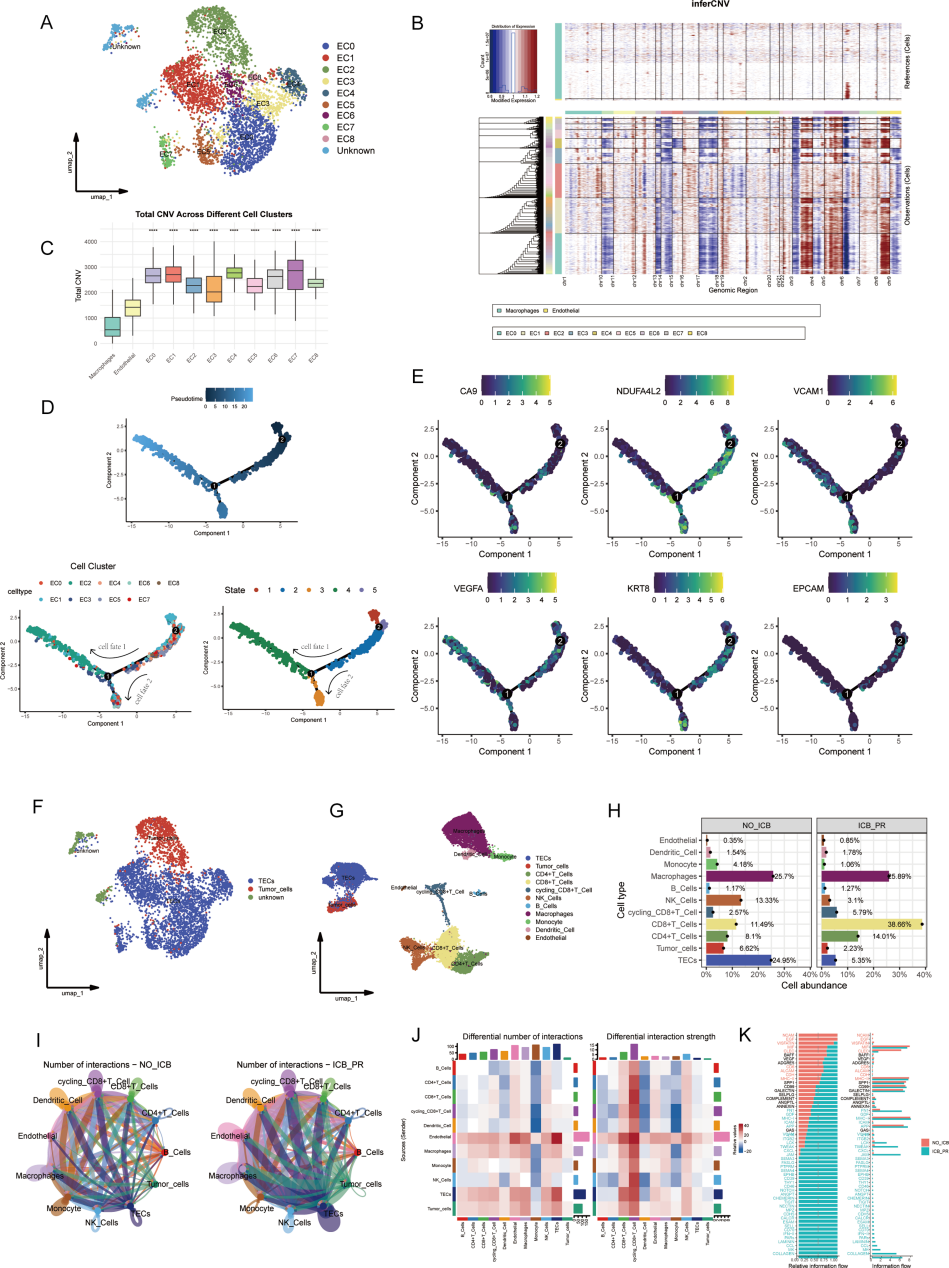
Malignant cell identification and analysis of cellular communication within the TME in ccRCC. **(A)** UMAP visualization identifying ten epithelial subclusters, comprising nine EC subclusters and one unknown subcluster. **(B)** A heatmap depicting CNV levels across 22 chromosomes for the nine EC subclusters, with macrophages and endothelial cells serving as references. Amplifications are marked in red and losses are indicated in blue. **(C)** A box plot illustrating total CNV levels; CNV was significantly higher in all EC clusters than in reference cells. **(D)** Analysis of differentiation trajectories within EC clusters; pseudotime and varying cellular states are displayed. **(E)** Dynamic expression patterns of cancer-related and epithelial marker genes along differentiation trajectories. **(F)** Annotation of malignant cells identified within the EC subclusters. **(G)** Updated UMAP showing revised cell annotations and the exclusion of unknown cells. **(H)** A bar plot representing the proportion of each cell cluster within different ICB treatment groups. **(I)** A circle plot illustrating variations in cell communication among different cell types within different ICB groups in ccRCC. **(J)** A heatmap detailing significant changes in interaction number and strength following ICB therapy. **(K)** Overview of information flow differences across various ICB treatment groups. ****p< 0.0001. TME, tumor microenvironment; ccRcc, clear cell renal cell carcinoma; UMAP, Uniform Manifold Approximation and Projection; EC, epithelial cell; CNV, copy number variation; ICB, immune checkpoint blockade.

All epithelial cells were categorized into subclusters, annotated as epithelial cells (ECs) or unidentified cells, based on classical markers for epithelial and immune cells (**Figure 2A**). The EC subclusters were subjected to an assessment of CNV levels across chromosomes to predict malignant transformation. The loss of chromosome 3p has been associated with tumor progression and the emergence of more aggressive ccRCC phenotypes (8, 9, 28). The results revealed that all EC subclusters exhibited significant CNV across the 23 chromosomes when compared with reference cells, indicative of a high likelihood of malignancy (**Figures 2B, C**). Additionally, pseudotime analysis was employed to infer cell differentiation trajectories based on gene expression patterns. This analysis pinpointed the EC0 and EC1 subclusters as origins of differentiation, with EC2 representing a terminal differentiation stage of cell fate 1. Throughout these trajectories, the EC subclusters progressed through five distinct states (**Figure 2D**). The expression levels of cancer-associated and epithelial marker genes along these trajectories served to identify the differentiated states of malignant cells. Notably, the cancer-related genes *CA9* and *NDUFA4L2* and the epithelial marker genes *KRT8* and *EPCAM* were expressed across cell states 1, 2, 3, and 5, and before the second differentiation node, marking the presence of TECs. Meanwhile, *VCAM1* and *VEGFA* were specifically expressed in cells of cluster EC2 and those of cell state 2, indicating that they were tumor cells (**Figures 2E, F**). In conclusion, there was a significant decrease in the proportions of tumor cells and TECs within the sample exhibiting a partial response to ICB therapy (**Figures 2G, H**). Additionally, significant heterogeneity was observed in the ccRCC TME.

### 3.3 Analysis of intercellular interactions within the ccRCC TME following ICB therapy

Our analysis of cell communication within the ccRCC TME post-ICB therapy unveiled significant alterations in the flow of information as well as in the number and intensity of intercellular interactions. As shown in the circular plot and heatmap in **Figures 2I, J**, there was a marked increase in the quantity and strength of interactions between tumor cells and TECs across all cell types within the ICB-PR group. Furthermore, we observed a substantial enhancement in communication among TECs, tumor cells, and both CD8+ and cycling CD8+ T cells in the ICB-PR group. Significant changes in information flow were primarily, but not exclusively, observed in the MHC-I, IL16, CD70, and CCL signaling pathways (**Figure 2K**). Following ICB therapy, malignant cells and CD8+ T cells emerged as the principal effector cells, exhibiting significantly strengthened intercellular interactions.

### 3.4 Transcriptional variability and prognostic significance of TUBA1C in ICB therapy

**Figure 3:**
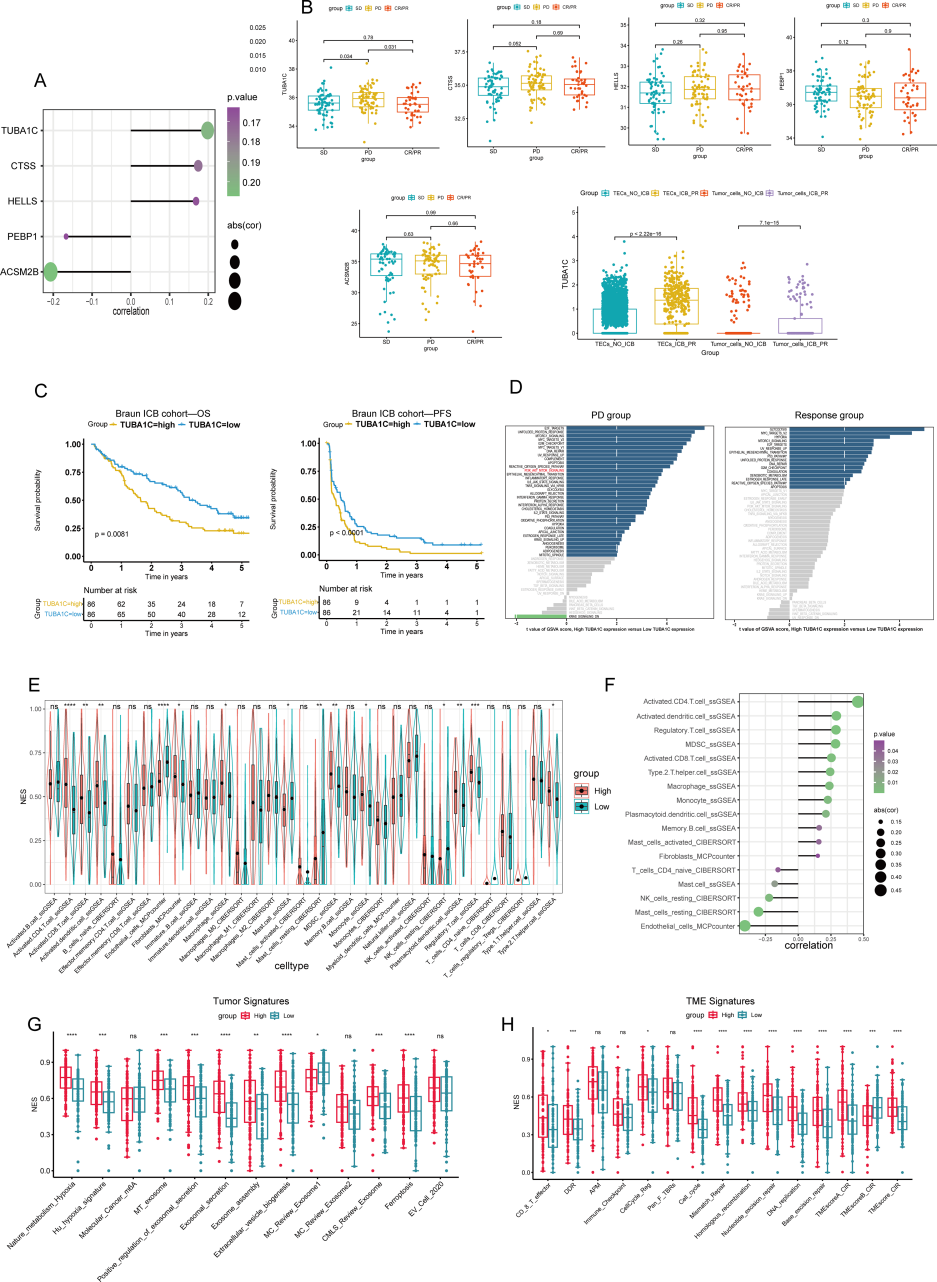
Analysis of TUBA1C expression and its impact on patient outcomes in the Braun ICB cohort. **(A)** A lollipop plot illustrating the relationship between five independent prognostic genes and CD274 expression. **(B)** Analysis of the mRNA expression levels of CD274-correlated genes across different response groups in the Braun ICB cohort, with a specific focus on TUBA1C expression in malignant cells from various ICB treatment groups. **(C)** Survival analysis demonstrating that high TUBA1C expression was associated with reduced OS and PFS. **(D)** Variations in hallmark molecular pathways across different response groups categorized by TUBA1C expression levels. **(E)** A comparison of immune cell infiltration levels, calculated using multiple algorithms, between two groups differentiated by TUBA1C expression. **(F)** Spearman correlation analysis evaluating the relationship between immune cell levels and TUBA1C expression. **(G, H)** ssGSEA-derived scores representing tumor and TME signatures across different TUBA1C expression groups. ****p< 0.0001, ***p<0.001, **p<0.01, *p<0.05. ICB, immune checkpoint blockade; OS, overall survival; PFS, progression-free survival; CD274, programmed death-ligand 1 (PD-L1); ssGSEA, single-sample gene set enrichment analysis; TME, tumor microenvironment; ns, not significant.

To investigate the transcriptional variability in malignant cells, we performed a differential gene expression analysis on tumor cells and TECs in both the ICB-PR and NO-ICB groups at the single-cell level. We identified 829 DEGs in tumor cells and TECs for further analysis. These DEGs were subjected to univariate Cox regression analysis, leading to the identification of 115 prognostic DEGs for further evaluation through multivariate Cox regression analyses within the Braun ICB cohort. Ultimately, 40 independently prognostic DEGs were delineated (**Supplementary Table 2**). Following a correlation analysis of the relationship between these 40 DEGs and CD274, only 5 of the DEGs presented a significant correlation with CD274, with TUBA1C demonstrating the most significant positive correlation (**Figure 3A**). We next explored the expression levels of the five candidate genes across the various response groups (PD, SD, CR/PR), and found that the expression of TUBA1C was significantly upregulated in the PD group when compared with that in both the SD and CR/PR groups. This pattern of heightened expression was also observed in tumor cells and TECs at the single-cell level following an ICB response, underscoring the pivotal role of TUBA1C in the TME post-therapy. No notable differences in the expression levels of the other four genes were observed among the response groups (**Figure 3B**). Subsequent analysis further indicated that patients with high TUBA1C expression had poorer OS and PFS (**Figure 3C**). To further investigate the potential pathways involved in ICB resistance and identify the underlying mechanisms, a gene set variation analysis (GSVA) was performed between the PD group and the response groups in the Braun ICB cohort. The results revealed that PI3K pathway activation was associated with elevated expression of PD-L1 in breast and prostate cancer cells, leading to immune evasion (29). Additionally, the Wnt and PI3K-related pathways have been shown to promote immune exclusion and dysfunction through the recruitment and differentiation of immunosuppressive cells in various cancer types (30). Here, we observed that the PI3K/AKT pathway was activated in the PD group, accompanied by high TUBA1C expression; however, no significant Wnt/β-catenin activity was detected. Meanwhile, the PI3K/AKT and Wnt/β-catenin pathways were not activated in the ICB response group (**Figure 3D**). Furthermore, patients in the high-TUBA1C-expression group exhibited enriched activity in oncogenic pathways, such as E2F targets, G2M checkpoint, p53 pathway, hypoxia, epithelial-mesenchymal transition (EMT), and DNA repair pathways in the two groups (**Figure 3D**). These findings highlight the potential mechanisms underlying the role of TUBA1C in carcinogenesis and immune dysfunction. Analysis of immune cell infiltration across the groups of the Braun ICB cohort revealed differential distributions of various immune cells. Notably, the group with elevated TUBA1C levels exhibited a significant increase in the abundance of activated CD4+ T cells, CD8+ T cells, macrophages, MDSCs, Tregs, and type 2 T helper (Th2) cells (**Figure 3E**). This trend was consistently observed across multiple analytical algorithms. Furthermore, a correlation analysis indicated that there was a positive association between TUBA1C expression and the abovementioned cell types. In contrast, resting NK cells, mast cells, and endothelial cells displayed a negative correlation with TUBA1C expression (**Figure 3F**). Our findings showed that patients with elevated TUBA1C expression displayed significantly higher tumor signature scores in several key areas, including hypoxia, exosomal secretion and assembly, extracellular vesicle biogenesis, and ferroptosis, among others (**Figure 3G**). Moreover, the scores for DNA damage response (DDR), antigen processing and presentation (APM), cell cycle regulation, and DNA replication, along with other TME signatures, were elevated in conjunction with increased TUBA1C expression (**Figure 3H**). These observations suggested that TUBA1C has significant malignancy potential in ccRCC.

### 3.5 Insights into TUBA1C in pan-cancer and pan-tissue: correlation, expression, and prognostic impact

**Figure 4:**
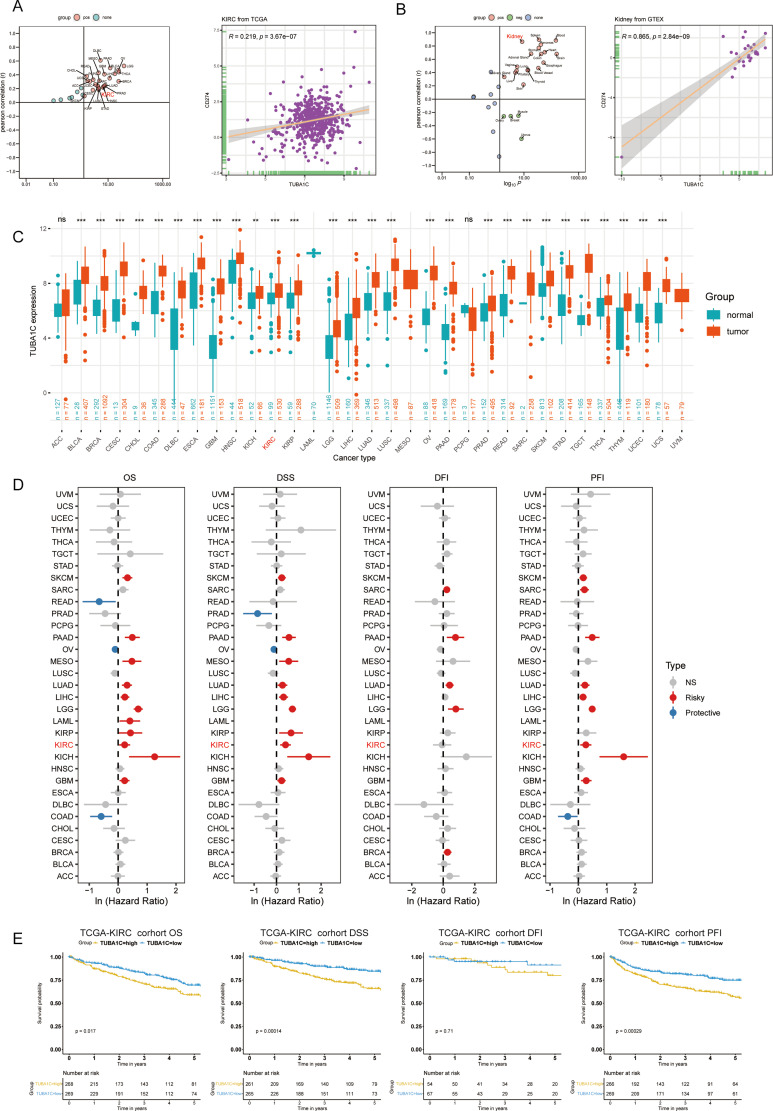
Pan-cancer and pan-tissue analysis of TUBA1C expression and its clinical implications. **(A)** A scatter plot showing the correlation between TUBA1C and CD274 across various cancers, highlighting a significant positive association in TCGA-KIRC cohort. **(B)** Pan-tissue analysis indicating a positive correlation between TUBA1C and CD274 in several normal tissues, particularly kidney tissue. **(C)** Integration of TCGA and GTEx datasets showing TUBA1C upregulation at the mRNA level across various cancer types. **(D)** Analysis depicting TUBA1C as a risk factor affecting prognosis (OS, DSS, DFI, and PFI) in various cancer types. **(E)** Survival analysis demonstrating that high TUBA1C expression is associated with poorer outcomes in terms of OS, DSS, and PFI in TCGA-KIRC cohort. TCGA, The Cancer Genome Atlas; KIRC, kidney renal clear cell carcinoma; GTEx, Genotype-Tissue Expression; OS, overall survival; DSS, disease-specific survival; DFI, disease-free interval; PFI, progression-free interval. ns, not significant. **p < 0.01, ***p < 0.001. ns, not significant.

To expand the understanding of the role of TUBA1C across a wide range of cancers and tissues, we conducted a comprehensive analysis focusing on its association with CD274, variations in gene expression, CNV, and methylation discrepancies, in addition to their implications for prognosis. Our analysis revealed that there was a significant positive association between TUBA1C and CD274 in several cancer types, including KIRC, kidney renal papillary cell carcinoma (KIRP), stomach adenocarcinoma (STAD), and breast invasive carcinoma (BRCA), among others (**Figure 4A**). This relationship was also evident in a variety of normal tissues, such as the kidney, liver, skin, and brain (**Figure 4B**), indicating that TUBA1C may play a pervasive role in contexts other than cancer. Further investigation showed that TUBA1C is not only highly expressed in KIRC but also in a broad spectrum of cancers (**Figure 4C**), suggesting that it has a wide-ranging oncogenic role. Furthermore, our analysis of CNV uncovered significant deletions or amplifications of TUBA1C in many cancer types. Notably, elevated TUBA1C expression displayed a significant positive correlation with CNV in ccRCC and lung adenocarcinoma (LUAD), among other cancer types (**Supplementary Figures 1A–C**). Additionally, we observed variations in methylation patterns across different regions of TUBA1C, including the transcription start site (TSS) and gene body, when compared with those seen in normal samples on a pan-cancer scale (**Supplementary Figure 1D**). While methylation discrepancies in several regions of TUBA1C were primarily associated with ccRCC, these variations influenced the expression level of TUBA1C in various cancer types (**Supplementary Figure 1E**). Importantly, the expression of TUBA1C significantly affected prognosis in various cancers, including ccRCC, LUAD, and pancreatic adenocarcinoma (PAAD) (**Figure 4D**). Specifically, in TCGA-KIRC cohort, we observed that TUBA1C expression markedly influenced the outcomes of ccRCC patients, including OS, PFS, and DPI (**Figure 4E**). The results of the pan-cancer and pan-tissue analysis underscored the critical role of TUBA1C in cancer biology and its potential as a prognostic marker in ccRCC. Our findings further highlighted the need for additional research into its mechanisms of action and their implications for cancer therapy.

### 3.6 Unraveling the role of TUBA1C: KEGG and GO enrichment analysis in TCGA-KIRC cohort

In this study, to delineate the functional implications of TUBA1C, we determined the correlation coefficients between TUBA1C and each gene within TCGA-KIRC cohort employing GSEA. Our findings indicated that genes that were positively correlated with TUBA1C were significantly involved in crucial biological pathways, including cell cycle regulation, DNA replication, and processes associated with lysosomes, ribosomes, and spliceosomes. Furthermore, TUBA1C was found to play a pivotal role in the biological processes of DNA replication, RNA splicing, and protein translation. On a cellular level, TUBA1C was found to contribute to the integrity of ribosomes, spliceosomes, and chromosome complexes. Regarding molecular function, TUBA1C demonstrated a strong association with mRNA transcription and protein translation (**Supplementary Figures 2A–D**).

### 3.7 The association between TUBA1C expression and clinical features

**Figure 5:**
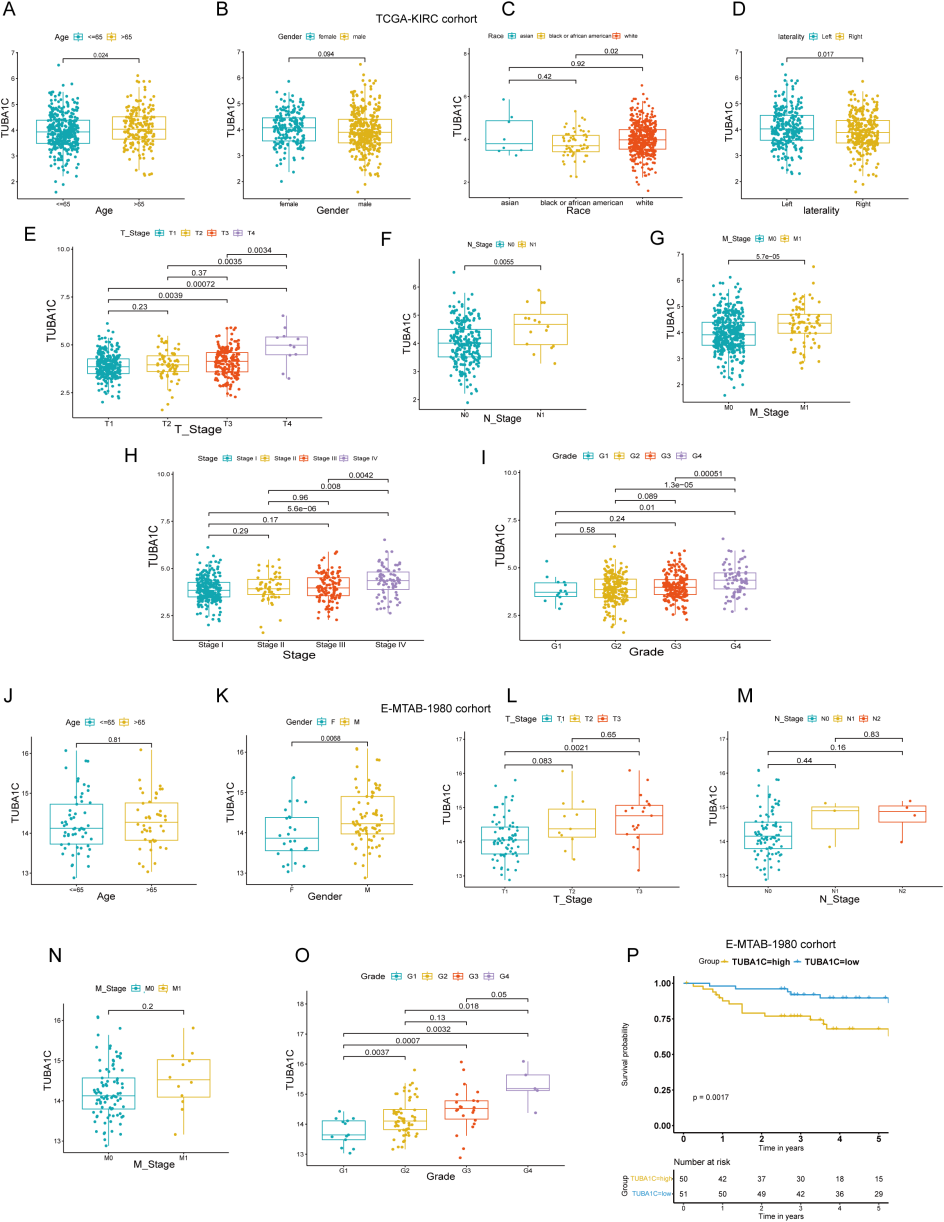
Correlation of TUBA1C expression with clinical characteristics in TCGA-KIRC and E-MTAB-1980 cohorts. **(A–I)** Analysis of the relationship between TUBA1C expression and various clinical parameters, including age, gender, race, laterality, TNM classification, tumor stage, and tumor grade in TCGA-KIRC cohort. **(J–O)** Examination of the correlation between TUBA1C expression and clinical features such as age, gender, TNM classification, tumor grade, and tumor stage in the E-MTAB-1980 cohort. **(P)** Survival analysis showing that high TUBA1C expression is associated with poorer outcomes in the E-MTAB-1980 cohort. TCGA, The Cancer Genome Atlas; KIRC, kidney renal clear cell carcinoma.

We investigated the expression levels of TUBA1C in various groups of differing clinical characteristics to elucidate the potential relationship between TUBA1C expression and clinical phenotypes. As shown in the box plots in **Figure 5A**, patients aged >65, those with left-side laterality, White patients, and patients at advanced T, N, M stages or higher grades exhibited significantly elevated TUBA1C expression levels compared with patients of other clinical phenotypes (**Figures 5A, C–I**). However, no significant association was found between TUBA1C expression and gender (**Figure 5B**). To validate these findings, the E-MTAB-1980 cohort was further examined. Consistent with our initial observations, the results showed that TUBA1C expression levels were significantly correlated with TNM stage, tumor grades, and tumor stage (**Figures 5J–O**). Moreover, elevated TUBA1C expression was demonstrated to be associated with poorer outcomes in the E-MTAB-1980 cohort (**Figure 5P**). Combined, the above results indicated that TUBA1C contributes to tumor progression, metastasis, and worse prognosis in patients with advanced ccRCC.

### 3.8 The effect of TUBA1C on the oncogenic and immune landscape in ccRCC

**Figure 6:**
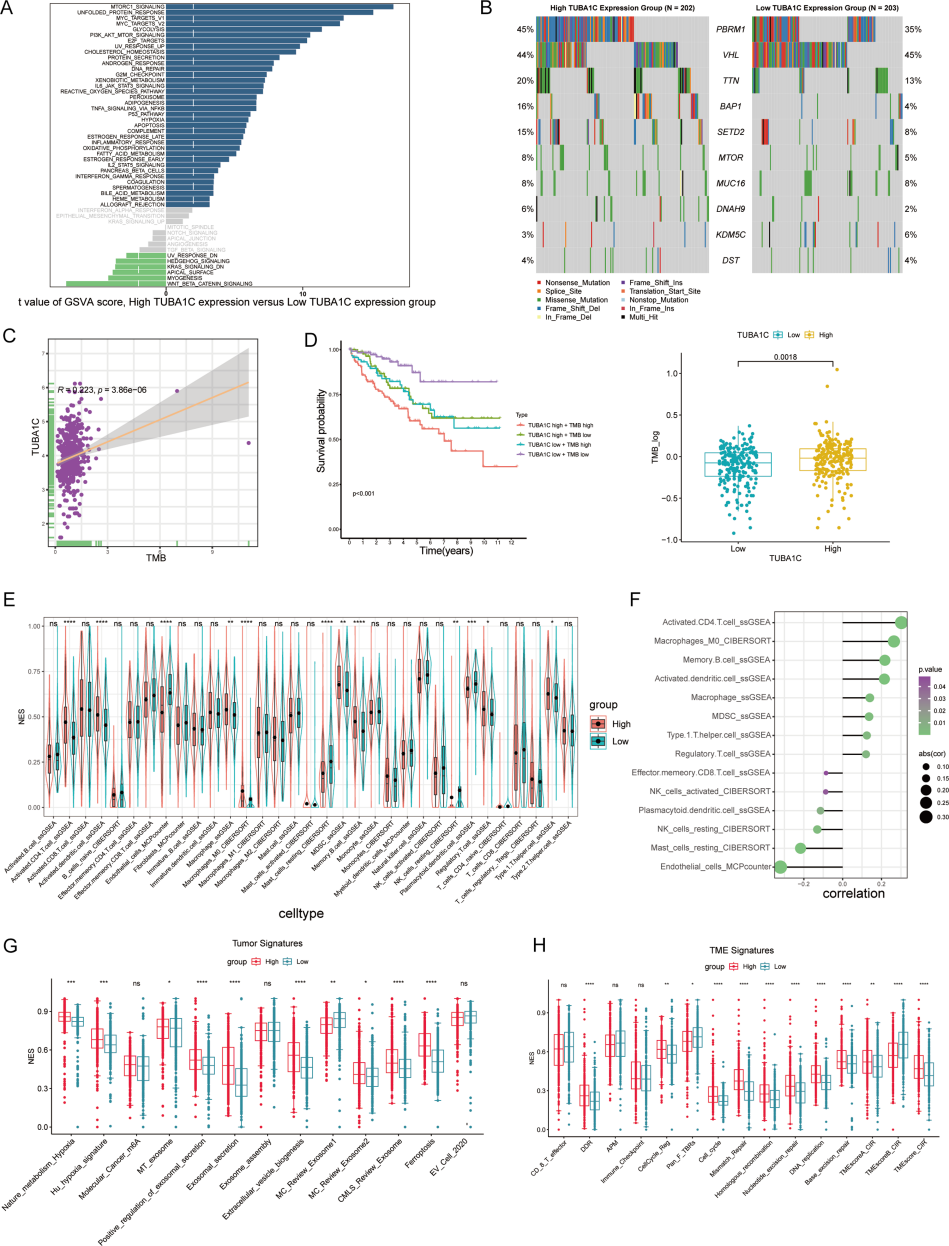
A comprehensive analysis of TUBA1C expression and its association with molecular pathways and clinical outcomes in ccRCC. **(A)** GSVA score distribution highlighting differences in pathway activation between high- and low-TUBA1C-expression groups across various hallmark pathways. **(B)** Mutation spectrum analysis comparing the top 10 genes in high- *versus* low-TUBA1C-expression groups within TCGA-KIRC cohort; the TMB was higher in the group with high TUBA1C expression. **(C)** A scatter plot showing the correlation between TUBA1C expression and the TMB, with a significant positive association being observed in TCGA-KIRC cohort. **(D)** Survival curves comparing overall survival among groups stratified by TUBA1C expression and TMB levels, underscoring the prognostic significance of both TUBA1C expression and the TMB. **(E, F)** An analysis of immune cell infiltration across different TUBA1C expression groups and the correlation between TUBA1C expression with immune cell profiles in TCGA-KIRC cohort. **(G, H)** Bar plots displaying the differential activity of tumor-related pathways and TME signatures between high- and low-TUBA1C-expression groups. ****p< 0.0001, ***p<0.001, **p<0.01, *p<0.05. ccRCC, clear cell renal cell carcinoma; GSVA, gene set variation analysis; TCGA, The Cancer Genome Atlas; KIRC, kidney renal clear cell carcinoma; TMB, tumor mutational burden; ns, not significant.

To further investigate the impact of differential TUBA1C expression levels on ccRCC patients who have not received ICB therapy, we conducted a comprehensive hallmark pathway analysis. We found that genes that were upregulated in the high-TUBA1C-expression group were significantly enriched in several oncogenic pathways, including MTORC1 signaling, MYC targets, PI3K/AKT signaling, E2F targets, G2M checkpoint, reactive oxygen species pathway, hypoxia, DNA repair, and the p53 pathway. Conversely, the upregulated genes in the low-TUBA1C-expression group were predominantly enriched in pathways such as “KRAS signaling down” and “Wnt/β-catenin signaling” (**Figure 6A**). We further downloaded somatic cell mutation data from TCGA database to calculate the TMB for each sample, with the results confirming that the group with high TUBA1C expression exhibited a higher TMB as well as greater genetic variation (**Figure 6B**). Moreover, correlation analysis demonstrated that a positive relationship existed between TUBA1C expression and the TMB within TCGA-KIRC cohort (**Figure 6C**). Notably, patients with high TUBA1C expression and TMB levels experienced poorer outcomes, whereas those with both low TUBA1C expression levels and a low TMB had the most favorable prognosis (**Figure 6D**). Lastly, utilizing multiple immune-related algorithms, we assessed all immune cell markers to map the immune microenvironment landscape induced by TUBA1C. The group with high TUBA1C expression in TCGA-KIRC cohort displayed a greater abundance of activated CD4+ T cells, activated dendritic cells, M0 macrophages, MDSCs, and Tregs, in line with the findings for the Braun ICB cohort (**Figures 3E**, **6E**). Similarly, a significant positive correlation was observed between TUBA1C expression and these immune cell populations (**Figure 6F**). Analysis of the tumor and TME signature scores in TCGA-KIRC cohort showed that, in line with that observed in the Braun ICB cohort, the group with elevated TUBA1C expression exhibited higher scores relating to carcinogenic pathways and TME activity (**Figures 3**, **6G, H**).

### 3.9 Dissecting the role of TUBA1C in immune regulation and drug sensitivity in ccRCC

**Figure 7:**
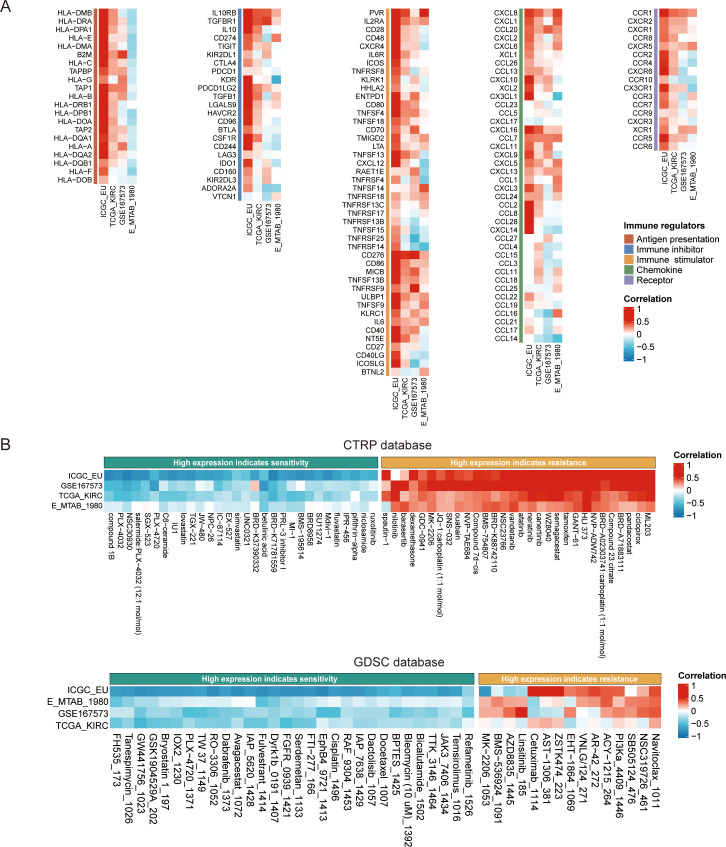
The impact of TUBA1C on immune modulation and drug sensitivity in ccRCC using the BEST database. **(A)** Correlation between TUBA1C expression and immune modulation modules across multiple datasets. The modules included antigen presentation machinery, immune inhibitors, stimulators, chemokines, and their receptors, highlighting the potential regulatory role of TUBA1C in the immune landscape of ccRCC. **(B)** Analysis of drug sensitivity variations associated with different expression levels of TUBA1C utilizing data from the CTRP and GDSC databases. Variation in TUBA1C expression was correlated with differential drug responsiveness; greater intensity of red indicates a stronger positive correlation and deeper blue signifies a stronger negative correlation. ccRCC, clear cell renal cell carcinoma; BEST, Biomarker Exploration of Solid Tumors; CTRP, Cancer Therapeutics Response Portal; GDSC: Genomics of Drug Sensitivity in Cancer.

We next explored the association between TUBA1C expression and immune regulation and drug sensitivity in ccRCC given its putative importance in personalized cancer therapy research. Our analysis, utilizing the BEST online tool, revealed the existence of a significant correlation between TUBA1C expression and various immune regulators in multiple ccRCC datasets. These regulators include key elements such as antigen presentation machinery, immune inhibitors, stimulators, chemokines, and receptors. Importantly, TUBA1C was found to be positively correlated with PDCD1, CD274, and CTLA4, which are therapeutic targets of ICB, in various datasets. The generated heatmaps, shown in **Figure 7A**, underscore these correlations, showcasing broadly consistent trends across the examined datasets and highlighting the potential role of TUBA1C in modulating the ccRCC immune landscape. Separately, we assessed the impact of TUBA1C expression on drug sensitivity employing data from the CTRP and GDSC databases. This analysis highlighted a distinct pattern, namely, that patients with higher levels of TUBA1C expression demonstrated resistance to several drugs, including afatinib, nilotinib, and PI3Ka_4409_1446. Conversely, these same patients showed increased sensitivity to other treatments such as ruxolitinib, refametinib, and JAK3_7406_1434 (**Figure 7B**). This variation in drug responses, based on TUBA1C expression levels, emphasizes the potential utility of TUBA1C as a predictive marker for therapeutic efficacy in ccRCC.

### 3.10 TUBA1C: a potential therapeutic target and its role in enhancing the response to ICB therapy in ccRCC

**Figure 8:**
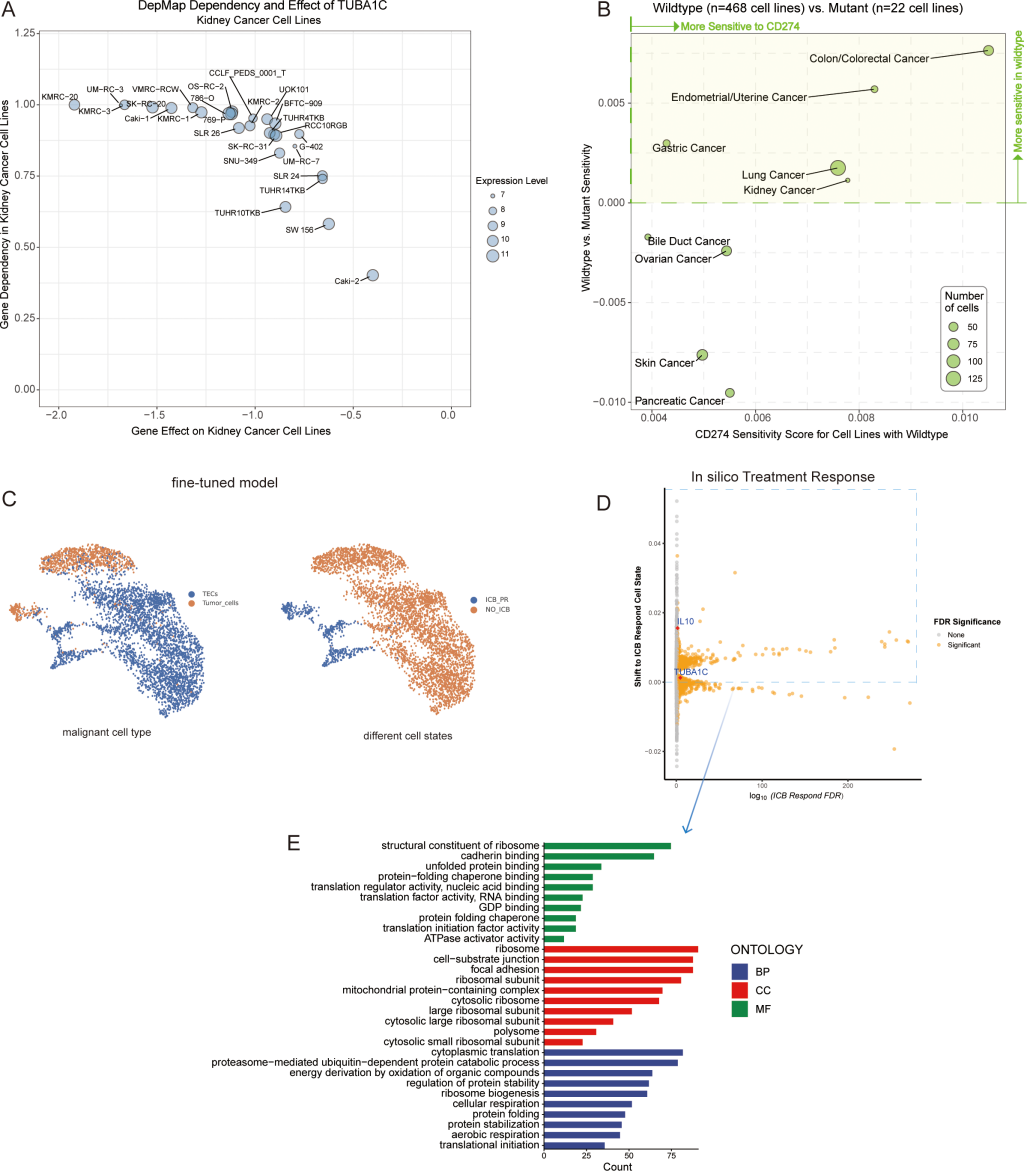
Analysis of TUBA1C gene function, mutation state, and impact on ICB resistance. **(A)** A dot plot displaying 26 kidney cancer cell lines characterized by TUBA1C expression, indicating a negative gene effect and a positive gene dependency on TUBA1C. **(B)** Analyzing the sensitivity of cancer cell lines with wild-type or mutated TUBA1C to anti-PD-L1 therapy, highlighting variations in drug responsiveness based on TUBA1C mutation status. **(C)** A fine-tuned Geneformer model was used to infer cell type between tumor cells and TECs among malignant cells (left). Geneformer distinguishes malignant cell states between the ICB-PR and NO-ICB groups from tumor cells and TECs by defining the embedding positions of each cell state. The NO-ICB group underwent *in silico* deletion analysis to identify genes whose removal resulted in a significant shift of the embedding towards the ICB-PR malignant cell state (right). **(D)** Visualization of gene effects determined through *in silico* knockout studies. Orange indicates significant candidate genes (FDR <0.05) that greatly influenced cell states post-knockout. **(E)** GO enrichment analysis in BP, CC, and MF for genes meeting a FDR threshold of <0.05. These genes were associated with positive shifts toward an ICB-responsive state. ICB, immune checkpoint blockade; PR, partial response; TEC, tumor-associated endothelial cells; GO, Gene Ontology; BP, Biological Process; CC, Cellular Component; MF, Molecular Function.

In our study, utilizing the DepMap database, we investigated the effects of TUBA1C knockout on the phenotypes of kidney cancer cell lines and examined the relationship between TUBA1C mutations and the efficacy of anti-PD-L1 therapy. As shown in the dot plot in **Figure 8A**, data analysis yielded a gene effect score of <0 for TUBA1C across 26 kidney cancer cell lines. This score signifies cell death or restricted growth following the CRISPR-Cas9-mediated knockout of TUBA1C, with all analyzed cell lines exhibiting varying degrees of dependence on TUBA1C for survival or growth (**Figure 8A**). Moreover, based on the mutation status of TUBA1C, we assessed the average dependency score for CD274, comparing wild-type (468 cell lines) and mutant categories (22 cell lines). Intriguingly, kidney cancer cell lines with wild-type TUBA1C displayed a higher CD274 dependency score than their mutant counterparts. This finding suggested that kidney cancer cell lines with wild-type TUBA1C are more susceptible to anti-PD-L1 therapy, and, conversely, that TUBA1C mutations may confer enhanced resistance to anti-PD-L1 therapy. The investigation was extended to include cell lines derived from lung cancer, gastric cancer, endometrial/uterine cancer, and colon/colorectal cancer (**Figure 8B**). These cancer types demonstrated a similar relationship between TUBA1C mutation and sensitivity to anti-PD-L1 therapy as that seen with the kidney cancer lines. These consistent outcomes across various cancer types highlighted the correlation between TUBA1C and CD274, positioning TUBA1C as a potential biomarker for targeted therapy.

We initially acquired the baseline Geneformer model and fine-tuned it over five epochs using our single-cell dataset. The optimized model demonstrated a noteworthy accuracy of 99.08% by the third epoch, and could effectively distinguish between different types of malignant cells and states. This was achieved using gene expression data as input and cell embeddings as output (**Figure 8C**). Building upon this refined model, we randomly selected 2,000 cells to perform *in silico* knockouts, aiming to observe changes in cell state post-gene knockout. Of the 13,881 genes knocked out *in silico*, 2,401 (FDR <0.05) were identified as having the potential to influence cell state subsequent to the knockout (**Supplementary Table 3**). Notably, knocking out TUBA1C was observed to induce a partial response state in malignant cells following ICB treatment. Interleukin 10 (*IL10*), a key gene implicated in ICB resistance across multiple studies (31, 32), served as a reference, exhibiting similar effects to TUBA1C following *in silico* knockout (**Figure 8D**). Interestingly, the genes that enhanced ICB treatment responses were predominantly associated with biological functions such as ribosome assembly, RNA binding, protein folding, and translation factor activity, which are significantly linked to TUBA1C gene function (**Figure 8E**). This provided strong evidence supporting a crucial role for TUBA1C as an oncogene across several types of cancer. In ccRCC, TUBA1C markedly influenced the proliferation of kidney cell lines. Furthermore, cell lines harboring mutated TUBA1C exhibited reduced sensitivity to anti-PD-L1 therapy relative to their wild-type counterparts. Notably, malignant cells demonstrated increased responsiveness to ICB therapy following TUBA1C knockout.

### 3.11 Expression patterns and clinical implications of TUBA1C and PD-L1 in ccRCC patients

**Figure 9:**
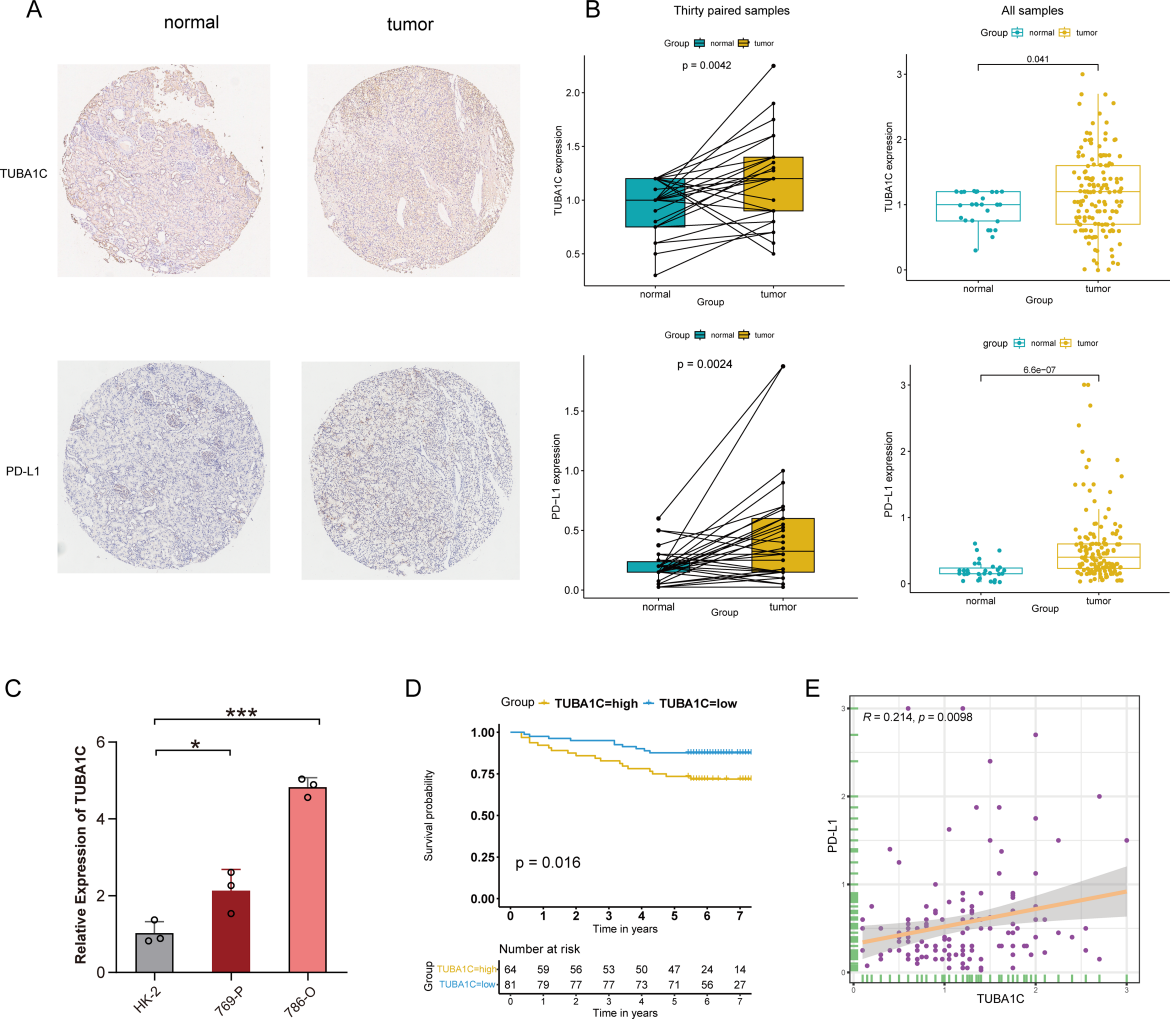
Immunohistochemical validation and clinical cohort analysis. **(A)** IHC staining showing the protein expression levels of TUBA1C and PD-L1 in normal *versus* tumor tissues from ccRCC patients. **(B)** Conducting differential expression analysis between samples from ccRCC patients and their matched adjacent non-tumor tissues. **(C)** Validation of the mRNA expression of TUBA1C in a human renal cell line and different ccRCC cell lines by RT-qPCR. ***p<0.001, *p<0.05. **(D)** Kaplan-Meier survival analysis illustrating the correlation between TUBA1C expression and OS in the clinical cohort. **(E)** Analysis of the correlation between the protein levels of TUBA1C and PD-L1 in ccRCC samples. IHC, immunohistochemistry; ccRCC, clear cell renal cell carcinoma; OS, overall survival.

After excluding invalid or deciduous slices from the TMA, robust TUBA1C and PD-L1 protein expression was observed in tissues from a clinical cohort of 145 out of 150 ccRCC patients and 30 patient-matched normal tissues using immunohistochemistry (**Figure 9A**). The patients were stratified into high- and low-expression groups based on the median expression levels of TUBA1C. Despite a lack of statistical significance in cancer grade, stage, and TNM classification between these groups, the group with high TUBA1C expression comprised a notably higher proportion of patients with advanced disease stages and grades (**Table 1**). A comparative analysis revealed that TUBA1C and PD-L1 protein expression was significantly higher in tumor tissues than in their normal counterparts, both paired and unpaired (**Figure 9B**). Additionally, the bioinformatics results were validated by RT-qPCR. We found that *TUBA1C* expression was significantly upregulated in 769-P and 786-O cells compared with that in HK-2 cells (**Figure 9C**). Moreover, survival analysis indicated that patients in the high-TUBA1C-expression group (**Figure 9D**) had poorer OS. Finally, our immunohistochemistry results demonstrated that there was a positive correlation between TUBA1C and PD-L1 expression at the protein level in patients with ccRCC (**Figure 9E**).

**Table 1:** Association between clinical characteristics and TUBA1C expression groups.

Characteristics	Variable	TUBA1C		All (n=145)	*P* value
		High expression (n=64)	Low expression (n=81)		
**Gender**	Female	15 (23.44%)	25 (30.86%)	40 (27.59%)	0.610
	Male	49 (76.56%)	56 (69.14%)	105 (72.41%)	
**Age**	<=65	46 (71.88%)	69 (85.19%)	115 (79.31%)	0.145
	>65	18 (28.12%)	12 (14.81%)	30 (20.69%)	
**T**	T1	52 (81.25%)	67 (82.72%)	119 (82.07%)	1
	T2	7 (10.94%)	8 (9.88%)	15 (10.34%)	
	T3	5 (7.81%)	6 (7.41%)	11 (7.59%)	
**N**	N0	63 (98.44%)	79 (97.53%)	142 (97.93%)	0.583
	N1	0	2 (2.47%)	2 (1.38%)	
	N2	1 (1.56%)	0	1 (0.69%)	
**M**	M0	64 (100.00%)	81 (100.00%)	145 (100.00%)	0
**Grade**	high	22 (34.38%)	20 (24.69%)	42 (28.97%)	0.443
	low	42 (65.62%)	61 (75.31%)	103 (71.03%)	
**Stage**	Stage 1	52 (81.25%)	67 (82.72%)	119 (82.07%)	0.972
	Stage 2	6 (9.38%)	8 (9.88%)	14 (9.66%)	
	Stage 3	5 (7.81%)	6 (7.41%)	11 (7.59%)	
	Stage 4	1 (1.56%)	0	1 (0.69%)	
**PD-L1**		0.67 ± 0.59	0.48 ± 0.50	0.56 ± 0.55	0.035

### 3.12 Functional validation of TUBA1C as an oncogene

**Figure 10:**
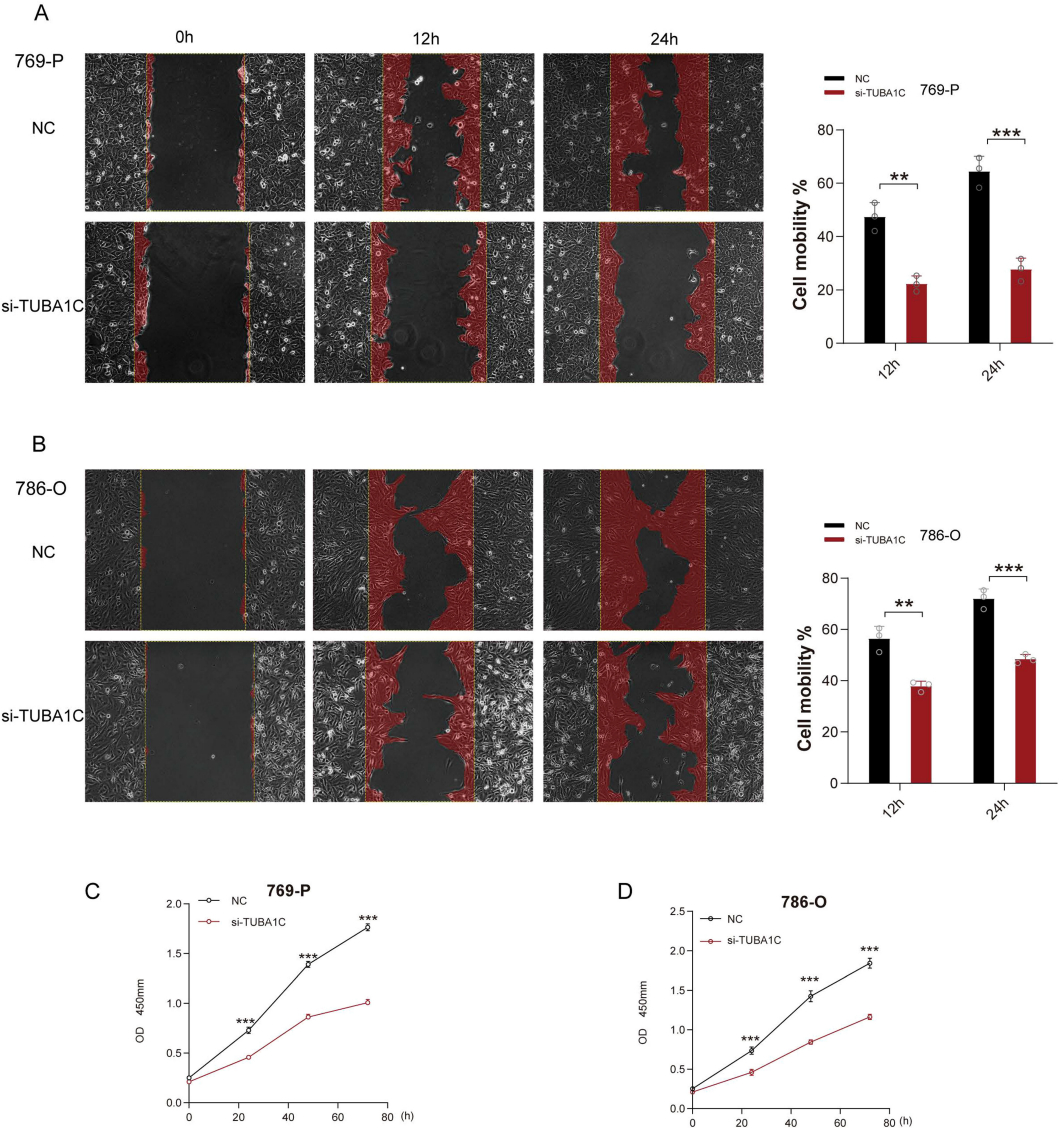
Functional validation of TUBA1C as an oncogene. **(A)** Wound healing assays performed on 769-P cancer cells exhibiting normal TUBA1C expression (NC) or with TUBA1C knockdown (si-TUBA1C) at 12 and 24 h are shown on the left. The bar plot on the right displays the quantified cell migration area at different time points. **(B)** Wound healing assays conducted on 786-O cells displaying normal TUBA1C expression (NC) or with TUBA1C knockdown (si-TUBA1C) at 12 and 24 h. ***p<0.001, **p<0.01. **(C, D)** Results of the CCK-8 assay in 769-P and 786-O cells after TUBA1C knockdown; significant reductions in cell viability and proliferation were observed.

Following the robust validation of expression, we next conduction an analysis of TUBA1C function. A wound healing assay revealed that the migratory potential of the ccRCC cell lines 769-P and 786-O were significantly inhibited at both 12 and 24 h post-TUBA1C knockdown (**Figures 10A, B**). Additionally, a CCK-8 assay demonstrated that both the viability and proliferative ability of these two cancer cell lines were markedly reduced following TUBA1C downregulation (**Figures 10C, D**). These results supported the bioinformatics analysis and suggested that TUBA1C promotes tumor progression and metastasis, thereby highlighting its potential as a prominent and effective biomarker in ccRCC.

## 4 Discussion

TUBA1C, an α-tubulin subtype, is crucial for the structure and function of microtubules, key components of the cytoskeleton. This multifunctional protein is involved in a broad range of cellular processes, including the regulation of cell division, the facilitation of intracellular transport, and the maintenance of cell morphology, in nearly all cell types (33, 34). Microtubule disturbances have been implicated in various types of cancers as well as numerous benign diseases. Consequently, microtubules have become prominent targets in cancer chemotherapy, and successful outcomes have been achieved with agents such as taxol derivatives and vinca alkaloids. However, these treatments often disrupt normal cellular functions and adversely affect healthy tissues (34). This highlights the need for therapeutic strategies that target microtubule-related anomalies more selectively, both in specific cancers and individual cancer cells, to minimize side effects. Recent studies have demonstrated that TUBA1C is upregulated in different types of cancers, including bladder cancer, breast cancer, and pancreatic ductal adenocarcinoma, and this upregulation is correlated with increased tumor progression and metastasis (16, 35–37). Despite these observations, the specific functions of TUBA1C in ccRCC remain poorly understood, as do the underlying mechanisms. Our study sought to address this knowledge gap, and found that TUBA1C plays a role in reshaping the tumor immune microenvironment and mediating resistance to ICB.

TUBA1C was found to be upregulated in malignant cells at the single-cell level following ICB therapy. The mRNA levels of TUBA1C were not only elevated in ccRCC samples in TCGA-KIRC cohort but were also markedly upregulated in patients within the PD group of the Braun ICB cohort. It has been shown in multiple ccRCC cohorts that increased expression of TUBA1C is associated with advanced tumor grade and stage, poorer prognosis, and shorter times to recurrence. Furthermore, TUBA1C is positively correlated with the TMB in ccRCC, indicating that TUBA1C participates in the occurrence and accumulation of mutations related to DNA repair and genome stability, thereby impacting outcomes and immune therapy responses in ccRCC patients (38). We identified a significant positive correlation between TUBA1C and CD274 at both the pan-cancer and pan-tissue levels, suggesting that TUBA1C has a strong and universal connection with CD274 under both physiological and pathological conditions. This relationship may influence the response to immune therapy.

Functional studies involving 26 kidney cancer cell lines demonstrated that TUBA1C is essential for their proliferation and survival. We also observed that kidney cancer cell lines harboring mutated TUBA1C exhibited greater resistance to anti-PD-L1 therapy compared with those with wild-type genetic configurations. Additionally, we employed a deep learning model to predict the impact of TUBA1C on the responses of malignant cells to ICB therapy. The results indicated that the *in silico* knockout of TUBA1C shifted the malignant cells towards a state of partial ICB response, mirroring the effects seen with IL10 knockout. Recent work has shown that IL-10 expression is upregulated in peritoneal metastatic lesions of both patients and mice resistant to ICB therapy in colorectal cancer (32). Additionally, several studies have confirmed that IL-10 release mediates ICB resistance in ovarian cancer (39, 40). Collectively, these findings underscore the important role of TUBA1C in inducing ICB resistance and its contribution to disease progression, highlighting its potential as a target to improve ICB responses and prognosis in ccRCC. The oncogenic function of TUBA1C was further validated by wound healing assays in two ccRCC cell lines, with the results revealing that the migratory ability of these cells was significantly reduced following the knockdown of TUBA1C. Similarly, the proliferative potential and the viability of the cells were significantly weakened, as estimated by the CCK-8 assay. Finally, using RT-qPCR, immunohistochemistry, and clinical cohort analysis, we confirmed that TUBA1C expression is upregulated at both the mRNA and protein levels in tumor tissue. Furthermore, PD-L1 was found to be positively correlated with TUBA1C expression in tumor tissues and co-localized on the cytomembrane of tumor cells. Although there were some discrepancies in the expression of TUBA1C between tumor stages and grades at the protein and mRNA levels, these differences can be attributed to the small sample sizes of advanced-stage and high-grade ccRCC patients in our clinical cohort. Importantly, patients with advanced disease often lack the opportunity for surgery, which limits the availability of tumor tissue samples for immunohistochemical experiments.

The TUBA1C protein, which is involved in the formation of mitotic spindles and is an integral constituent of the cytoskeleton, has been demonstrated to play a pivotal role in the regulation of key biological processes such as the cell cycle, DNA replication, RNA splicing, and protein translation (34). These roles were substantiated through a GSEA of gene sets from the KEGG and GO databases, thereby highlighting the significant influence of TUBA1C on cell cycle regulation, RNA transcription, and protein synthesis in ccRCC. Importantly, dysregulation of the cell cycle serves as a primary catalyst for the uncontrolled proliferation typical of cancer cells, independently of growth-stimulating signals. This is further exacerbated by continuous damage to DNA that occurs during cell division and in response to various external stimuli, which disrupts cell cycle regulation and promotes tumorigenesis (41, 42). Central to numerous DNA-dependent processes, the maintenance of chromosomal protein complex structure governs chromosome segregation during mitosis, transcriptional control, and the DNA damage response, including replication, repair, and recombination (43). Disruptions in cell cycle regulation may lead to oxidative stress, DNA mutations, and metabolic remodeling, culminating in unchecked cell proliferation and tumorigenesis (44). Overall, our findings underscore the oncogenic nature of the TUBA1C protein. A GSVA indicated that elevated TUBA1C expression was correlated with enhanced activity of oncogenic pathways such as DNA repair, E2F targets, G2/M checkpoint, p53 signaling, hypoxia, MYC targets, PI3K/AKT pathway, and oxidative phosphorylation; this further implies that patients with elevated TUBA1C expression exhibit notable DNA damage and cell cycle dysregulation, which alters metabolism within the TME in ccRCC. Additionally, hypoxic conditions prevalent in substantial tumor masses promote the progression of ccRCC, as supported by data from the Braun ICB and TCGA-KIRC cohorts, thus confirming the multifaceted role of TUBA1C in this malignancy (45, 46).

Regarding the state of immune cell infiltration under different ICB exposure conditions, consistency in the changes observed can reflect the TUBA1C-mediated recruitment of immune cells and reduce the impact of redundancy factors. Multiple algorithms assessing immune cell abundance demonstrated consistent trends across different TUBA1C expression groups. In both the Braun ICB and TCGA-KIRC cohorts, patients exhibited high proportions of activated CD4+ T cells, Tregs, MDSCs, and macrophages. In contrast, the abundance of NK cells and mast cells showed a negative correlation with TUBA1C expression, and there was no significant relationship between TUBA1C and CD8+ T cells. A recent study indicated that CD4+ T cells facilitate the activation of a gene expression program in CD8+ T cells, enhancing the cytotoxic activity of CD8+ T lymphocytes through varied molecular mechanisms (47). High levels of myeloid cell infiltration are associated with poorer prognosis and ICB resistance following anti-PD-L1 treatment in RCC (48). Additionally, MDSCs, characterized as highly heterogeneous and immature myeloid cells, exert an immunosuppressive effect that promotes tumor invasion and supports immune escape (49). MDSCs can reduce NK cell activity, regulate Treg differentiation, and induce an immunosuppressive phenotype in macrophages (6). Furthermore, M2 macrophages can suppress the activation of CD8+ T cells and promote the recruitment of Tregs, which contributes to immune evasion (50). These findings highlight the potential role of TUBA1C in contributing to resistance to ICB treatment. Meanwhile, increased Treg infiltration levels are associated with a poorer prognosis, inhibiting an effective anti-tumor immune response and promoting distant metastasis (51, 52). Incomplete Treg depletion induces compensatory proliferation after ICB exposure and the upregulation of TIM-3 and LAG-3 can induce Treg-driven ICB resistance (6). In various solid tumors, the abundance of Tregs engenders a worse prognosis and impairs the response to ICB treatment (18, 53). This strongly supports that TUBA1C recruits MDSCs and Tregs via the PI3K/AKT pathway, inducing an immunosuppressive phenotype in macrophages and dysfunction in CD8+ T cells, thereby reshaping the immunosuppressive tumor microenvironment and mediating ICB resistance in ccRCC.

We identified TUBA1C as a key determinant of the immunosuppressive microenvironment of ccRCC and found that it was significantly associated with tumor progression, poor prognosis, and resistance to ICB therapy. This renders TUBA1C a promising therapeutic target whose suppression can potentially reverse the immunosuppressive tumor microenvironment, delay cancer progression, improve the response to ICB treatment, and prolong the survival of ccRCC patients. Despite the importance of our findings, this study had several limitations. All the cohorts were retrospective and the sample sizes were limited. Additionally, we did not perform *in vivo* animal experiments to investigate the function of TUBA1C in reshaping the tumor microenvironment, carcinogenesis, and resistance to ICB therapy as well as the underlying mechanisms. These limitations will be addressed in future studies. To strengthen our findings, we plan to expand the sample size and incorporate prospective data. Multi-omics analyses, including scRNA-seq, spatial RNA sequencing, and bulk RNA sequencing, will be integrated to validate the composition and co-localization relationships among major cell types, and the potential mechanisms will be further validated. This comprehensive approach will provide deeper insights into the role of TUBA1C and its therapeutic potential in ccRCC. Future research should also focus on the clinical application of TUBA1C inhibitors or modulators, potentially in combination with existing ICB therapies. Understanding the interaction between TUBA1C and other immune-modulatory pathways could reveal new strategies for enhancing anti-tumor immunity. Addressing these areas will be vital for translating our findings into clinical practice, ultimately improving outcomes for ccRCC patients.

## Data Availability

The original contributions presented in the study are included in the article/**Supplementary Material**. Further inquiries can be directed to the corresponding author.

## References

[1] Barata PC Rini BI. Treatment of renal cell carcinoma: Current status and future directions. CA: Cancer J Clin. (2017) 67:507–24. doi: 10.3322/caac.21411 28961310

[2] Siegel RL Miller KD Fuchs HE Jemal A. Cancer statistics, 2022. CA: Cancer J Clin. (2022) 72:7–33. doi: 10.3322/caac.21708 35020204

[3] Dabestani S Marconi L Hofmann F Stewart F Lam TB Canfield SE. Local treatments for metastases of renal cell carcinoma: a systematic review. Lancet Oncol. (2014) 15:e549–61. doi: 10.1016/S1470-2045(14)70235-9 25439697

[4] Sung H Ferlay J Siegel RL Laversanne M Soerjomataram I Jemal A. Global cancer statistics 2020: GLOBOCAN estimates of incidence and mortality worldwide for 36 cancers in 185 countries. CA: Cancer J Clin. (2021) 71:209–49. doi: 10.3322/caac.21660 33538338

[5] Krishna C DiNatale RG Kuo F Srivastava RM Vuong L Chowell D. Single-cell sequencing links multiregional immune landscapes and tissue-resident T cells in ccRCC to tumor topology and therapy efficacy. Cancer Cell. (2021) 39:662–77 e6. doi: 10.1016/j.ccell.2021.03.007 33861994 PMC8268947

[6] Morad G Helmink BA Sharma P Wargo JA. Hallmarks of response, resistance, and toxicity to immune checkpoint blockade. Cell. (2021) 184:5309–37. doi: 10.1016/j.cell.2021.09.020 PMC8767569 34624224

[7] Cha JH Chan LC Li CW Hsu JL Hung MC. Mechanisms controlling PD-L1 expression in cancer. Mol Cell. (2019) 76:359–70. doi: 10.1016/j.molcel.2019.09.030 PMC6981282 31668929

[8] Miao D Margolis CA Gao W Voss MH Li W Martini DJ. Genomic correlates of response to immune checkpoint therapies in clear cell renal cell carcinoma. Sci (New York NY). (2018) 359:801–6. doi: 10.1126/science.aan5951 PMC6035749 29301960

[9] Bi K He MX Bakouny Z Kanodia A Napolitano S Wu J. Tumor and immune reprogramming during immunotherapy in advanced renal cell carcinoma. Cancer Cell. (2021) 39:649–61 e5. doi: 10.1016/j.ccell.2021.02.015 33711272 PMC8115394

[10] Hanahan D Weinberg RA. Hallmarks of cancer: the next generation. Cell. (2011) 144:646–74. doi: 10.1016/j.cell.2011.02.013 21376230

[11] Rini BI Campbell SC Escudier B. Renal cell carcinoma. Lancet (London England). (2009) 373:1119–32. doi: 10.1016/S0140-6736(09)60229-4 19269025

[12] Hegde PS Chen DS. Top 10 challenges in cancer immunotherapy. Immunity. (2020) 52:17–35. doi: 10.1016/j.immuni.2019.12.011 31940268

[13] Rooney MS Shukla SA Wu CJ Getz G Hacohen N. Molecular and genetic properties of tumors associated with local immune cytolytic activity. Cell. (2015) 160:48–61. doi: 10.1016/j.cell.2014.12.033 25594174 PMC4856474

[14] Bagchi S Yuan R Engleman EG. Immune checkpoint inhibitors for the treatment of cancer: clinical impact and mechanisms of response and resistance. Annu Rev Pathol. (2021) 16:223–49. doi: 10.1146/annurev-pathol-042020-042741 33197221

[15] Bian T Zheng M Jiang D Liu J Sun H Li X. Prognostic biomarker TUBA1C is correlated to immune cell infiltration in the tumor microenvironment of lung adenocarcinoma. Cancer Cell Int. (2021) 21:144. doi: 10.1186/s12935-021-01849-4 33653340 PMC7923461

[16] Jiang Y Zhu C Huang H Huang G Fu B Xi X. TUBA1C is a potential new prognostic biomarker and promotes bladder urothelial carcinoma progression by regulating the cell cycle. BMC cancer. (2023) 23:716. doi: 10.1186/s12885-023-11209-2 37528357 PMC10391756

[17] Wang H Cui H Yang X Peng L. TUBA1C: a new potential target of LncRNA EGFR-AS1 promotes gastric cancer progression. BMC cancer. (2023) 23:258. doi: 10.1186/s12885-023-10707-7 36941595 PMC10026485

[18] Sharma P Goswami S Raychaudhuri D Siddiqui BA Singh P Nagarajan A. Immune checkpoint therapy-current perspectives and future directions. Cell. (2023) 186:1652–69. doi: 10.1016/j.cell.2023.03.006 37059068

[19] Braun DA Hou Y Bakouny Z Ficial M Sant’ Angelo M Forman J. Interplay of somatic alterations and immune infiltration modulates response to PD-1 blockade in advanced clear cell renal cell carcinoma. Nat Med. (2020) 26:909–18. doi: 10.1038/s41591-020-0839-y PMC7499153 32472114

[20] Zhang J Liu F Guo W Bi X Yuan S Shayiti F. Single-cell transcriptome sequencing reveals aberrantly activated inter-tumor cell signaling pathways in the development of clear cell renal cell carcinoma. J Transl Med. (2024) 22:37. doi: 10.1186/s12967-023-04818-9 38191424 PMC10775677

[21] Wu SZ Al-Eryani G Roden DL Junankar S Harvey K Andersson A. A single-cell and spatially resolved atlas of human breast cancers. Nat Genet. (2021) 53:1334–47. doi: 10.1038/s41588-021-00911-1 PMC9044823 34493872

[22] Qiu X Mao Q Tang Y Wang L Chawla R Pliner HA. Reversed graph embedding resolves complex single-cell trajectories. Nat Methods. (2017) 14:979–82. doi: 10.1038/nmeth.4402 PMC5764547 28825705

[23] Jin S Plikus MV Nie Q. CellChat for systematic analysis of cell-cell communication from single-cell and spatially resolved transcriptomics. (2023). 2023.11.05.565674. Available online at: https://www.biorxiv.org/content/10.1101/2023.11.05.565674v1 10.1038/s41596-024-01045-4 39289562

[24] Charoentong P Finotello F Angelova M Mayer C Efremova M Rieder D. Pan-cancer immunogenomic analyses reveal genotype-immunophenotype relationships and predictors of response to checkpoint blockade. Cell Rep. (2017) 18:248–62. doi: 10.1016/j.celrep.2016.12.019 28052254

[25] Liu Z Liu L Weng S Xu H Xing Z Ren Y. BEST: a web application for comprehensive biomarker exploration on large-scale data in solid tumors. J Big Data. (2023) 10:165. doi: 10.1186/s40537-023-00844-y

[26] Theodoris CV Xiao L Chopra A Chaffin MD Al Sayed ZR Hill MC. Transfer learning enables predictions in network biology. Nature. (2023) 618:616–24. doi: 10.1038/s41586-023-06139-9 PMC10949956 37258680

[27] Vaswani A Shazeer N Parmar N Uszkoreit J Jones L Gomez AN. Attention is all you need. (2017) 30. Available online at: https://arxiv.org/abs/1706.03762.

[28] Li Y Lih TM Dhanasekaran SM Mannan R Chen L Cieslik M. Histopathologic and proteogenomic heterogeneity reveals features of clear cell renal cell carcinoma aggressiveness. Cancer Cell. (2023) 41:139–63 e17. doi: 10.1016/j.ccell.2022.12.001 36563681 PMC9839644

[29] Crane CA Panner A Murray JC Wilson SP Xu H Chen L. PI(3) kinase is associated with a mechanism of immunoresistance in breast and prostate cancer. Oncogene. (2009) 28:306–12. doi: 10.1038/onc.2008.384 PMC3786571 18850006

[30] Luke JJ Bao R Sweis RF Spranger S Gajewski TF. WNT/β-catenin pathway activation correlates with immune exclusion across human cancers. Clin Cancer Res. (2019) 25:3074–83. doi: 10.1158/1078-0432.CCR-18-1942 PMC6522301 30635339

[31] Alcantara MB Tang WS Wang D Kaniowski D Kang E Dizman N. Targeting STAT3 in tumor-associated antigen-presenting cells as a strategy for kidney and bladder cancer immunotherapy. Front Immunol. (2023) 14:1274781. doi: 10.3389/fimmu.2023.1274781 38259453 PMC10800835

[32] Küçükköse E Heesters BA Villaudy J Verheem A Cercel M van Hal S. Modeling resistance of colorectal peritoneal metastases to immune checkpoint blockade in humanized mice. J Immunother Cancer. (2022) 10:e005345. doi: 10.1136/jitc-2022-005345 36543378 PMC9772695

[33] Ko P Choi JH Song S Keum S Jeong J Hwang YE. Microtubule acetylation controls MDA-MB-231 breast cancer cell invasion through the modulation of endoplasmic reticulum stress. Int J Mol Sci. (2021) 22:6018. doi: 10.3390/ijms22116018 34199510 PMC8199658

[34] Borisy G Heald R Howard J Janke C Musacchio A Nogales E. Microtubules: 50 years on from the discovery of tubulin. Nat Rev Mol Cell Biol. (2016) 17:322–8. doi: 10.1038/nrm.2016.45 PMC5116387 27103327

[35] Gui S Chen P Liu Y Chen Q Cheng T Lv S. TUBA1C expression promotes proliferation by regulating the cell cycle and indicates poor prognosis in glioma. Biochem Biophys Res Commun. (2021) 577:130–8. doi: 10.1016/j.bbrc.2021.08.079 34517210

[36] Lou W Ding B Zhong G Yao J Fan W Fu P. RP11-480I12.5-004 promotes growth and tumorigenesis of breast cancer by relieving miR-29c-3p-mediated AKT3 and CDK6 degradation. Mol Ther Nucleic Acids. (2020) 21:916–31. doi: 10.1016/j.omtn.2020.07.022 PMC7452110 32810693

[37] Albahde MAH Zhang P Zhang Q Li G Wang W. Upregulated expression of TUBA1C predicts poor prognosis and promotes oncogenesis in pancreatic ductal adenocarcinoma via regulating the cell cycle. Front Oncol. (2020) 10:49. doi: 10.3389/fonc.2020.00049 32117719 PMC7033491

[38] Jardim DL Goodman A de Melo Gagliato D Kurzrock R. The challenges of tumor mutational burden as an immunotherapy biomarker. Cancer Cell. (2021) 39:154–73. doi: 10.1016/j.ccell.2020.10.001 PMC7878292 33125859

[39] Luo Y Shreeder B Jenkins JW Shi H Lamichhane P Zhou K. Th17-inducing dendritic cell vaccines stimulate effective CD4 T cell-dependent antitumor immunity in ovarian cancer that overcomes resistance to immune checkpoint blockade. J Immunother Cancer. (2023) 11:e007661. doi: 10.1136/jitc-2023-007661 37918918 PMC10626769

[40] Lamichhane P Karyampudi L Shreeder B Krempski J Bahr D Daum J. IL10 release upon PD-1 blockade sustains immunosuppression in ovarian cancer. Cancer Res. (2017) 77:6667–78. doi: 10.1158/0008-5472.CAN-17-0740 PMC5712245 28993412

[41] Massagué J. G1 cell-cycle control and cancer. Nature. (2004) 432:298–306. doi: 10.1038/nature03094 15549091

[42] Molinari M. Cell cycle checkpoints and their inactivation in human cancer. Cell Prolif. (2000) 33:261–74. doi: 10.1046/j.1365-2184.2000.00191.x PMC6496592 11063129

[43] Hoencamp C Rowland BD. Genome control by SMC complexes. Nat Rev Mol Cell Biol. (2023) 24:633–50. doi: 10.1038/s41580-023-00609-8 37231112

[44] Liu J Peng Y Wei W. Cell cycle on the crossroad of tumorigenesis and cancer therapy. Trends Cell Biol. (2022) 32:30–44. doi: 10.1016/j.tcb.2021.07.001 34304958 PMC8688170

[45] Jing X Yang F Shao C Wei K Xie M Shen H. Role of hypoxia in cancer therapy by regulating the tumor microenvironment. Mol cancer. (2019) 18:157. doi: 10.1186/s12943-019-1089-9 31711497 PMC6844052

[46] Schödel J Grampp S Maher ER Moch H Ratcliffe PJ Russo P. Hypoxia, hypoxia-inducible transcription factors, and renal cancer. Eur Urol. (2016) 69:646–57. doi: 10.1016/j.eururo.2015.08.007 PMC5012644 26298207

[47] Borst J Ahrends T Bąbała N Melief CJM Kastenmüller W. CD4+ T cell help in cancer immunology and immunotherapy. Nat Rev Immunol. (2018) 18:635–47. doi: 10.1038/s41577-018-0044-0 30057419

[48] McDermott DF Huseni MA Atkins MB Motzer RJ Rini BI Escudier B. Clinical activity and molecular correlates of response to atezolizumab alone or in combination with bevacizumab versus sunitinib in renal cell carcinoma. Nat Med. (2018) 24:749–57. doi: 10.1038/s41591-018-0053-3 PMC6721896 29867230

[49] Li K Shi H Zhang B Ou X Ma Q Chen Y. Myeloid-derived suppressor cells as immunosuppressive regulators and therapeutic targets in cancer. Signal Transduct Target Ther. (2021) 6:362. doi: 10.1038/s41392-021-00670-9 34620838 PMC8497485

[50] Xue J Schmidt SV Sander J Draffehn A Krebs W Quester I. Transcriptome-based network analysis reveals a spectrum model of human macrophage activation. Immunity. (2014) 40:274–88. doi: 10.1016/j.immuni.2014.01.006 PMC3991396 24530056

[51] Reticker-Flynn NE Zhang W Belk JA Basto PA Escalante NK Pilarowski GOW. Lymph node colonization induces tumor-immune tolerance to promote distant metastasis. Cell. (2022) 185:1924–42.e23. doi: 10.1016/j.cell.2022.04.019 35525247 PMC9149144

[52] Tanaka A Sakaguchi S. Regulatory T cells in cancer immunotherapy. Cell Res. (2017) 27:109–18. doi: 10.1038/cr.2016.151 PMC5223231 27995907

[53] Fu J Xu D Liu Z Shi M Zhao P Fu B. Increased regulatory T cells correlate with CD8 T-cell impairment and poor survival in hepatocellular carcinoma patients. Gastroenterology. (2007) 132:2328–39. doi: 10.1053/j.gastro.2007.03.102 17570208

