# Supplementary material for: TUBA1C orchestrates the immunosuppressive tumor microenvironment and resistance to immune checkpoint blockade in clear cell renal cell carcinoma
Source: Front Immunol. 2024 Sep 5;15:1457691. doi: 10.3389/fimmu.2024.1457691 (PMC11410638; doi:10.3389/fimmu.2024.1457691)
Supplement: Supplementary file 1 [file DataSheet1.docx]

Supplementary Material

TUBA1C Orchestrates the Immunosuppressive Tumor Microenvironment and Resistance to Immune Checkpoint Blockade in Clear Cell Renal Cell Carcinoma

**Junyi Li1 †, Meixue Chen2 †, Ming Tong1, Qingfei Cao1***

^1^ Department of Urology, The First Affiliated Hospital of Jinzhou Medical University, Jinzhou, Liaoning, China

^2^ Department of Pediatric, The First Affiliated Hospital of Jinzhou Medical University, Jinzhou, Liaoning, China

^3^ First Clinical College, Jinzhou Medical University, Jinzhou, Liaoning, China

*** Correspondence:**

Qingfei Cao^1^

caoqingfei@jzmu.edu.cn

# Supplementary Figures and Tables

## Supplementary Figures

**Supplementary Figure 1.** Comprehensive Pan-Cancer and Pan-Tissue Analysis of TUBA1C Focusing on CNV and Methylation Patterns. **(A)** Overview of TUBA1C copy number variations across multiple cancer types. **(B-C)** Correlation analysis between TUBA1C expression and its CNV across various cancers, with a detailed focus on ccRCC. **(D)** Differential analysis of TUBA1C methylation patterns between normal and tumor samples across different cancer types. **(E)** Investigative analysis of the relationship between TUBA1C expression levels and methylation status. ***p<0.001; **p<0.01; *p<0.05. Abbreviations: CNV, copy number variation.

**Supplementary Figure 2.** Functional Characterization of TUBA1C.**(A)** Top five KEGG pathways influenced by TUBA1C. **(B)** Top five GO BP associated with TUBA1C. **(C)** Top five GO CC involving TUBA1C. **(D)** Top five GO MF linked to TUBA1C. Abbreviations: KEGG, Kyoto Encyclopedia of Genes and Genomes; GO, Gene Ontology; BP, biological process; CC, cellular component; MF, molecular function; GSEA, Gene Set Enrichment Analysis.

## Supplementary Table

**Supplementary Table 1.** Primer and siRNA Sequences. This table provides the sequences of primers used for RT-qPCR and siRNA used in plasmid transfection in this study.

**Supplementary Table 2.** Gene list from univariate and multivariate Cox regression analysis in the Braun cohort.

**Supplementary Table 3.** In Silico Knockout Analysis of Geneformer.
