# Supplementary figures and images for: TUBA1C orchestrates the immunosuppressive tumor microenvironment and resistance to immune checkpoint blockade in clear cell renal cell carcinoma
Source: Front Immunol. 2024 Sep 5;15:1457691. doi: 10.3389/fimmu.2024.1457691 (PMC11410638; doi:10.3389/fimmu.2024.1457691)

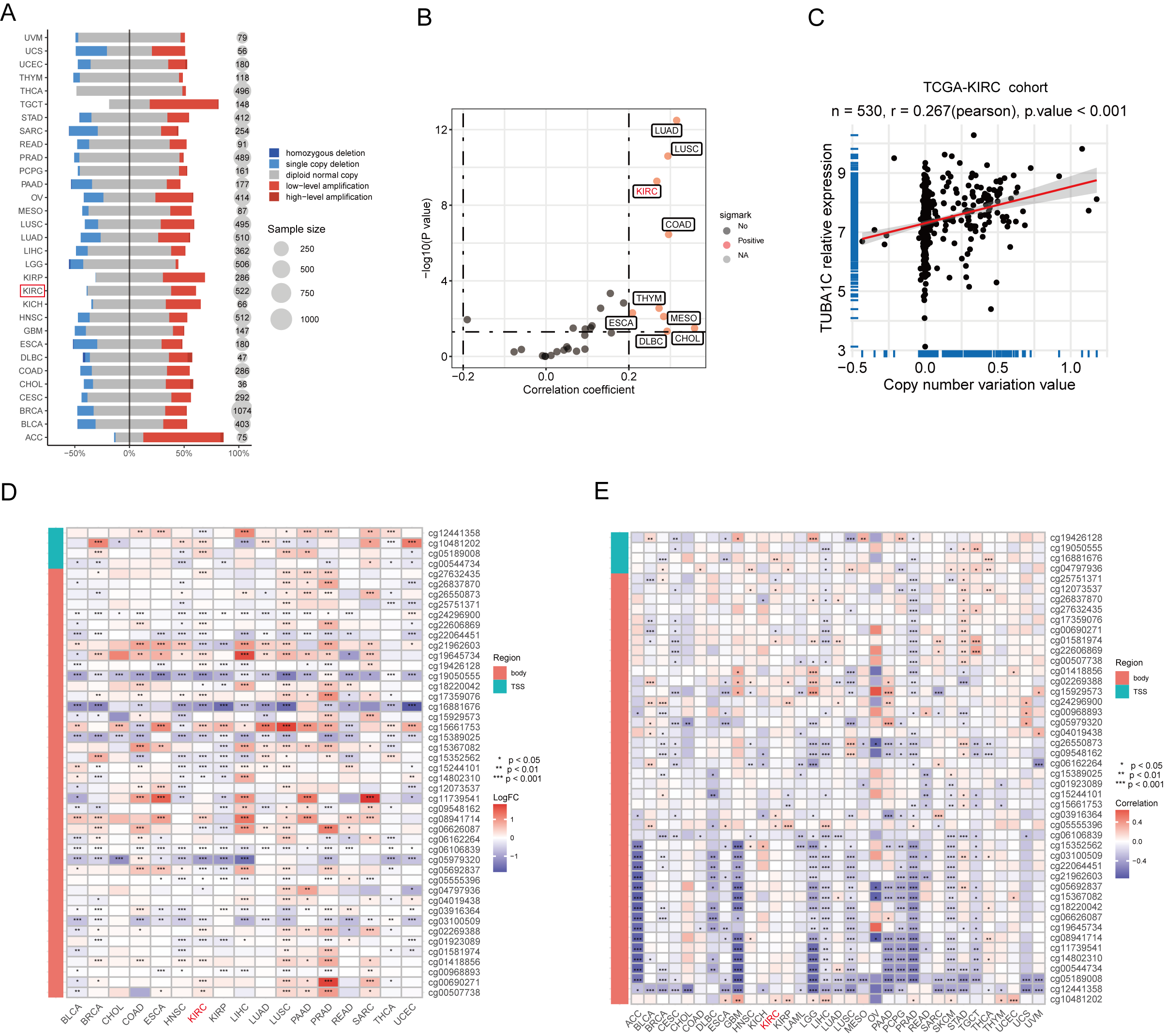

Supplement: Supplementary file 2 [file Image1.tif]

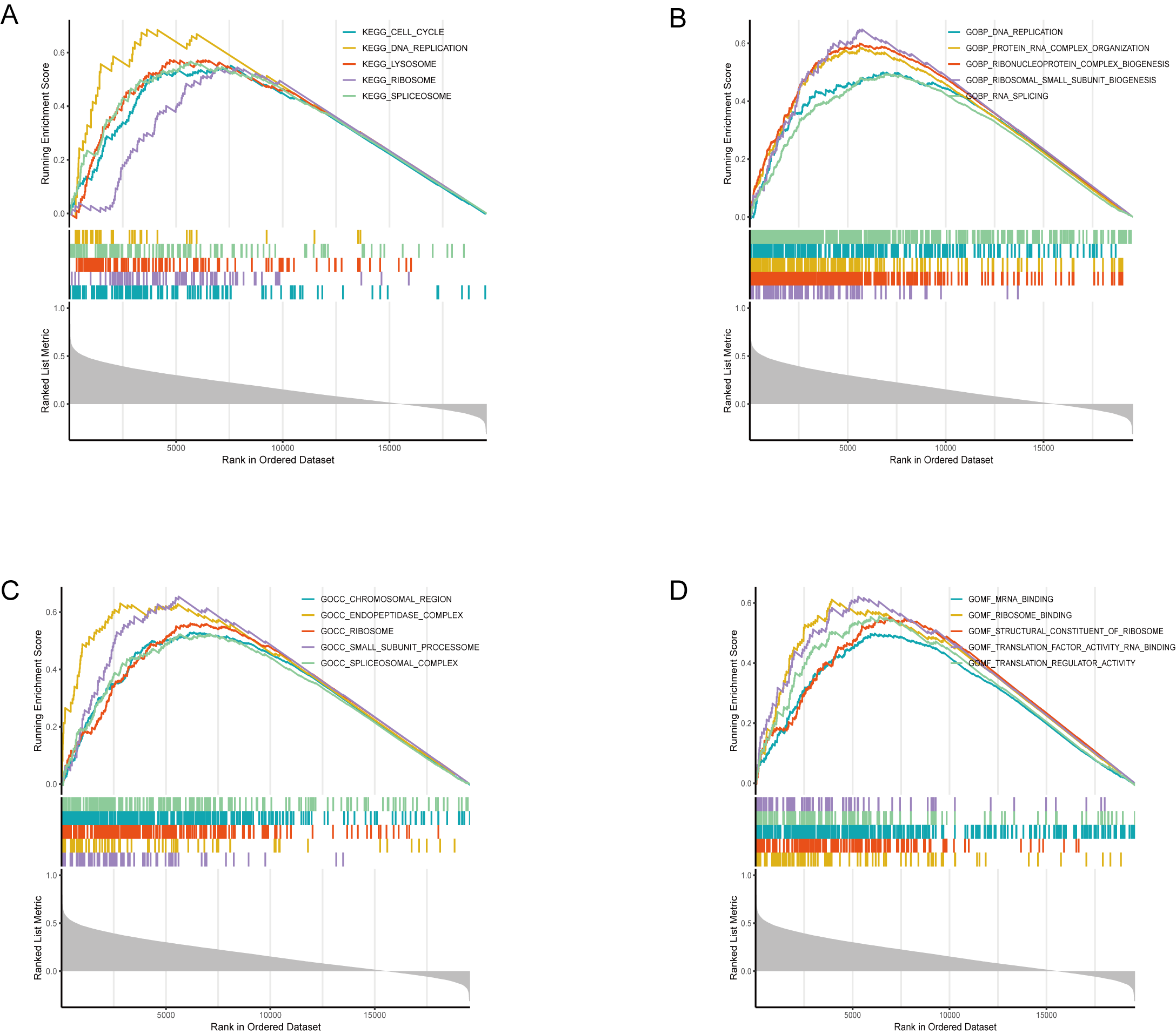

Supplement: Supplementary file 3 [file Image2.tif]
